# Supplementary material for: The GATA Transcription Factor Gaf1 Represses tRNAs, Inhibits Growth, and Extends Chronological Lifespan Downstream of Fission Yeast TORC1
Source: Cell Rep. 2020 Mar 10;30(10):3240–3249.e4. doi: 10.1016/j.celrep.2020.02.058 (PMC7068653; doi:10.1016/j.celrep.2020.02.058)
Supplement: Document S1. Figures S1–S5 and Table S1 [file mmc1.pdf]

Cell Reports, Volume *30*

Supplemental Information

The GATA Transcription Factor Gaf1 Represses tRNAs, Inhibits Growth, and Extends Chronological Lifespan Downstream of Fission Yeast TORC1

María Rodríguez-López, Suam Gonzalez, Olivia Hillson, Edward Tunnacliffe, Sandra Codlin, Victor A. Tallada, Jürg Bähler, and Charalampos Rallis

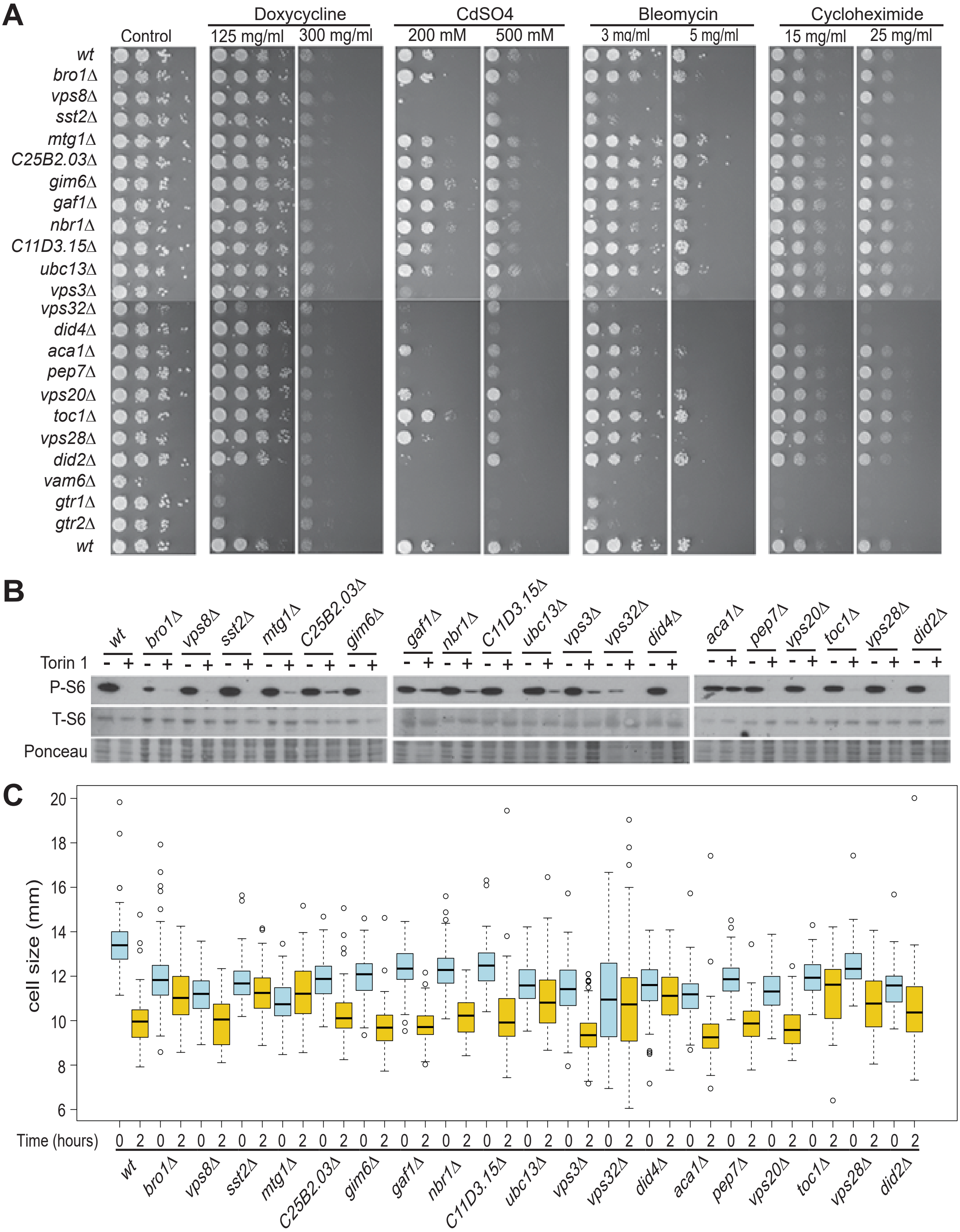

Figure S1. Characterization of Torin1-resistant strains, Related to Figure 1.
A. Resistance to Torin1 is not a result of multi-drug resistance. Spot assays of wild-type and the 19 resistant mutants in presence of different drugs as indicated. Control mutants that show multi-drug sensitivity are also spotted (*vam6Δ*, *gtr1Δ*, *gtr2Δ*) to show that the drugs are functional.
B. Phosphorylation status of ribosomal S6 protein in verified Torin1-resistant mutants in the presence or absence of Torin1 as indicated. **C.** Cell size upon division of wild-type and resistant mutant cells, before (blue) and after (yellow) Torin1 treatment.

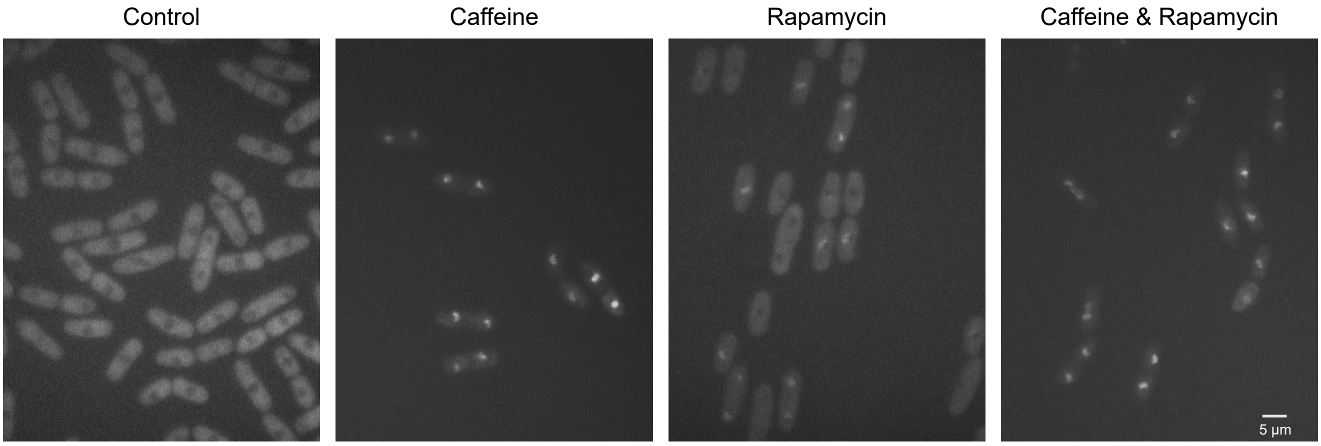

Figure S2. Fluorescence microscopy of cells expressing GFP-tagged Gaf1, Related to Figure 3.
Cells were grown in YES liquid medium to mid-exponential phase (OD600 =0.5), when caffeine (10 mM), rapamycin (100 ng/ml), or both drugs combined at same doses were added, and cells were incubated with shaking at 32°C for 30 minutes. Control cells were not exposed to the drugs but otherwise treated the same. Cells were photographed in a Zeiss Axioskop microscope using the Volocity software. All cells showed nuclear localization of Gaf1 after treatment with caffeine or with both drugs; the nuclear translocation of Gaf1 was less efficient in cells treated with rapamycin only, reflecting that rapamycin alone only partially inhibits TORC1 in *S. pombe* (Rallis et al., 2013).

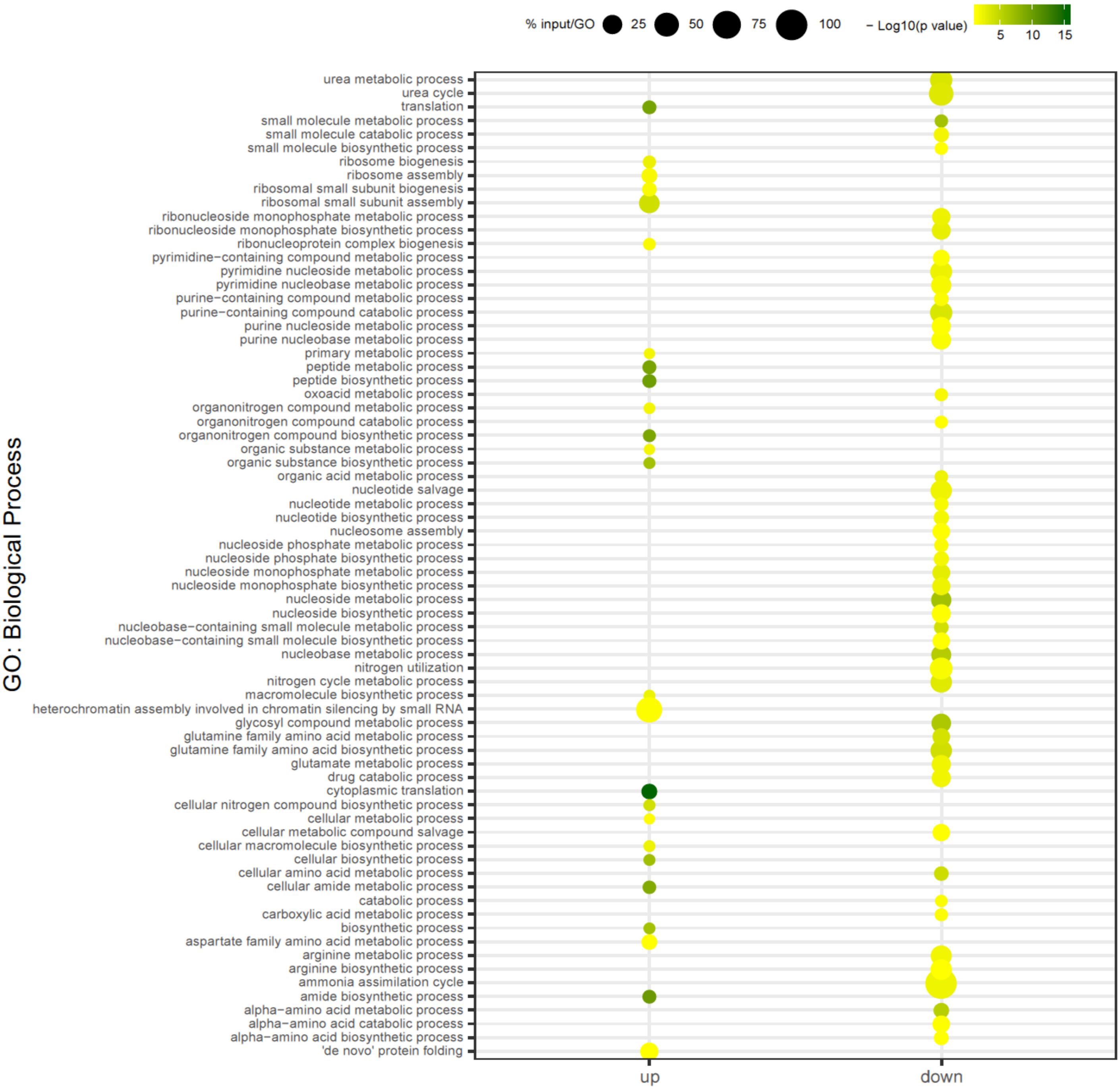

Figure S3. Functional enrichments among differentially expressed genes, Related to Figure 3. Visualization of all GO Biological Process categories enriched among the 108 genes that are repressed (down) and the 90 genes that are induced (up). Gene enrichment analysis was done using g:profiler (https://academic.oup.com/nar/advance-article/doi/10.1093/nar/gkz369/5486750). Results were plotted using a custom R script. Colors represent -log10 p values for the GO term enrichments. The size of the bubbles represents the ratio between the number of genes in the gene list and the total number of genes in the GO category (in percentage); only terms with p values <0.05 are represented.

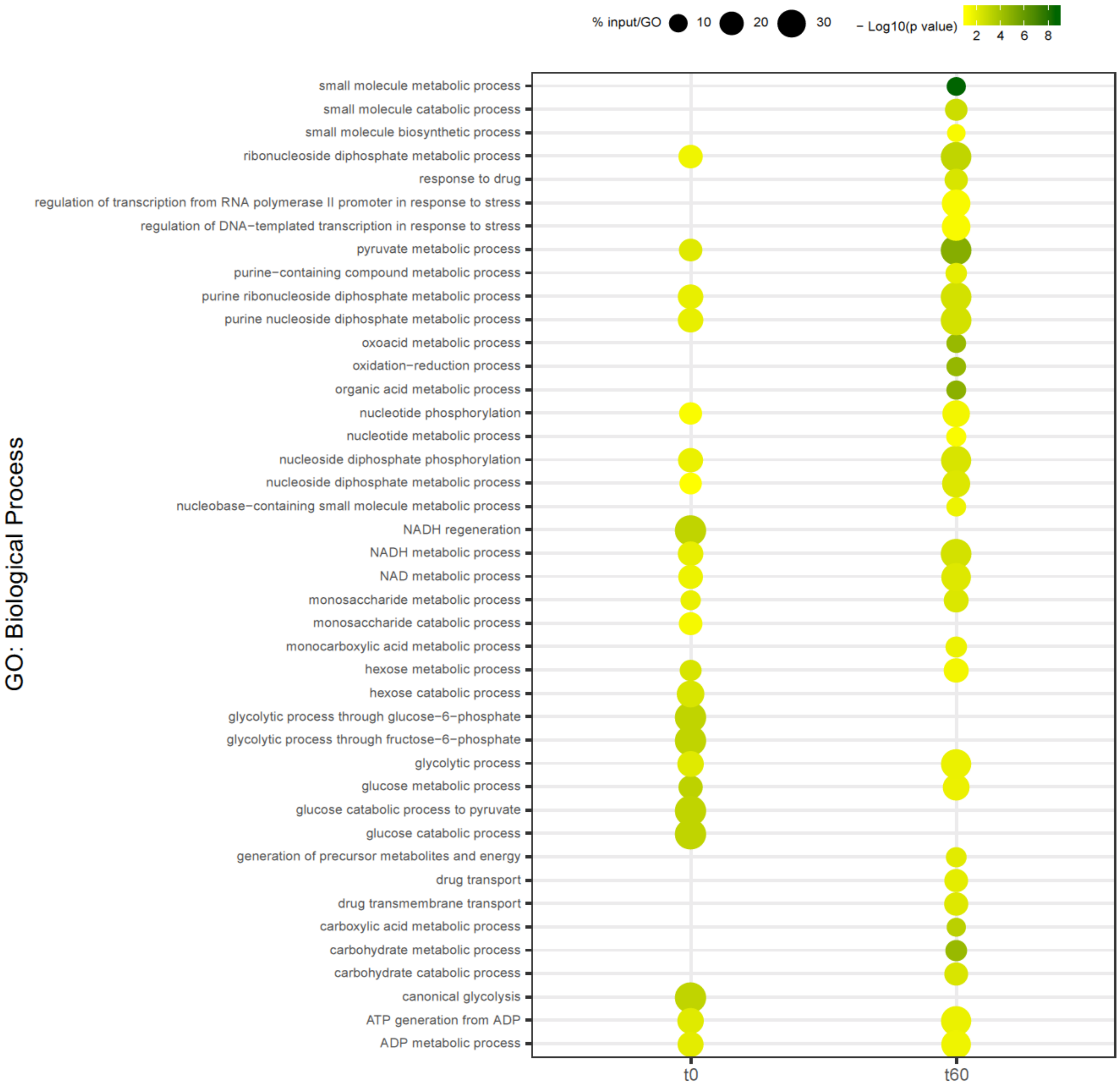

Figure S4. Functional enrichments among Gaf1-target genes, Related to Figure 4.
Visualization of all GO Biological Process categories enriched among the 245 protein-coding genes whose promoters are bound by Gaf1 after 60 min of Torin1 treatment. See Figure S3 for details.

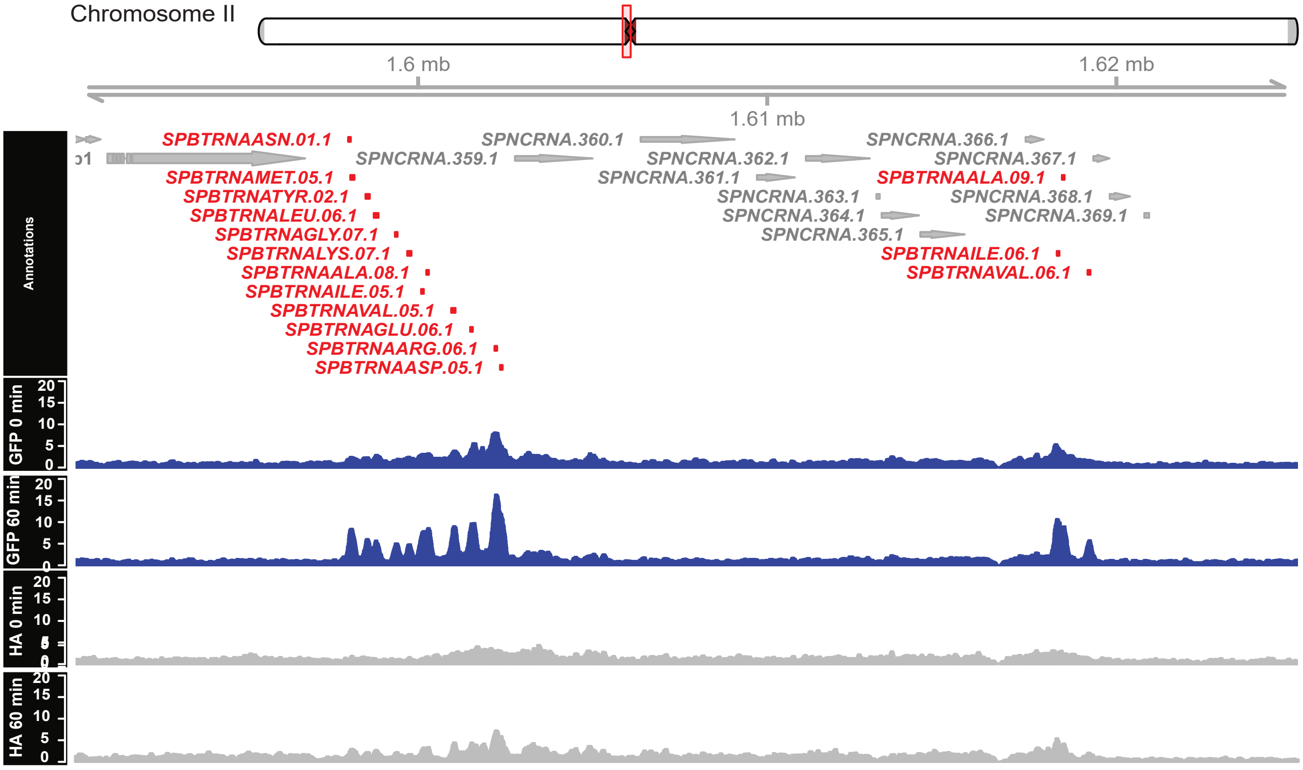

Figure S5. Gaf1 binding at tRNA genes, Related to Figure 4C.
Gaf1 binding peaks within Chromosome II region containing clustered tRNA genes (marked in red). Binding profiles are shown for cells before (0 min) and 60 min after treatment with Torin 1 as indicated. Signals from experimental IPs (GFP) are shown in blue, while control IPs (HA) are shown in grey.

Table S1. Torin1-resistant mutants, Related to Figure 1.
List of the 19 mutants that show resistance to Torin1. Mutants with black fonts have previously been shown to be resistant to Torin1. Mutants with red fonts are first reported as Torin1-resistant in this study.

SYSTEMATIC ID	GENE NAME	PRODUCT DESCRIPTION
SPAC21E11.04	*aca1*	L-azetidine-2-carboxylic acid acetyltransferase Aca1
SPAC17G6.05c	*bro1*	BRO1 domain protein Bro1 (predicted)
SPBC13G1.12	*did2*	ESCRT III complex subunit Did2 (predicted)
SPAC4F8.01	*did4*	ESCRT III complex subunit Did4
SPCC1902.01	*gaf1*	transcription factor Gaf1
SPBC1D7.01	*gim6*	prefoldin subunit 1 (predicted)
SPBC25B2.04c	*mtg1*	mitochondrial translation factor (GTPase) Mtg1 (predicted)
SPBP35G2.11c	*nbr1*	cargo receptor for selective autophagy pathway
SPAC17G6.08	*pep7*	prevacuole/endosomal FYVE tethering component Pep7 (predicted)
SPAC19B12.10	*sst2*	human AMSH/STAMBP protein homolog, ubiquitin specific-protease
SPBP18G5.03	*toc1*	Tor complex Tor2 interacting protein 1
SPAC11E3.04c	*ubc13*	ubiquitin conjugating enzyme E2 Ubc13
SPBC215.14c	*vps20*	ESCRT III complex subunit Vps20
SPAC1B3.07c	*vps28*	ESCRT I complex subunit Vps28
SPCC364.05	*vps3*	CORVET complex subunit, GTPase regulator Vps3 (predicted)
SPAC1142.07c	*vps32*	ESCRT III complex subunit Vps32
SPAC17A2.06c	*vps8*	CORVET complex WD repeat Vps8 (predicted)
SPAC11D3.15		5-oxoprolinase (ATP-hydrolizing) (predicted)
SPBC25B2.03		zf-C3HC4 type zinc finger
