# Supplementary material for: The GATA Transcription Factor Gaf1 Represses tRNAs, Inhibits Growth, and Extends Chronological Lifespan Downstream of Fission Yeast TORC1
Source: Cell Rep. 2020 Mar 10;30(10):3240–3249.e4. doi: 10.1016/j.celrep.2020.02.058 (PMC7068653; doi:10.1016/j.celrep.2020.02.058)
Supplement: Document S2. Article plus Supplemental Information [file mmc4.pdf]

Cell Reports

Report

The GATA Transcription Factor Gaf1 Represses tRNAs, Inhibits Growth, and Extends Chronological Lifespan Downstream of Fission Yeast TORC1

Graphical Abstract

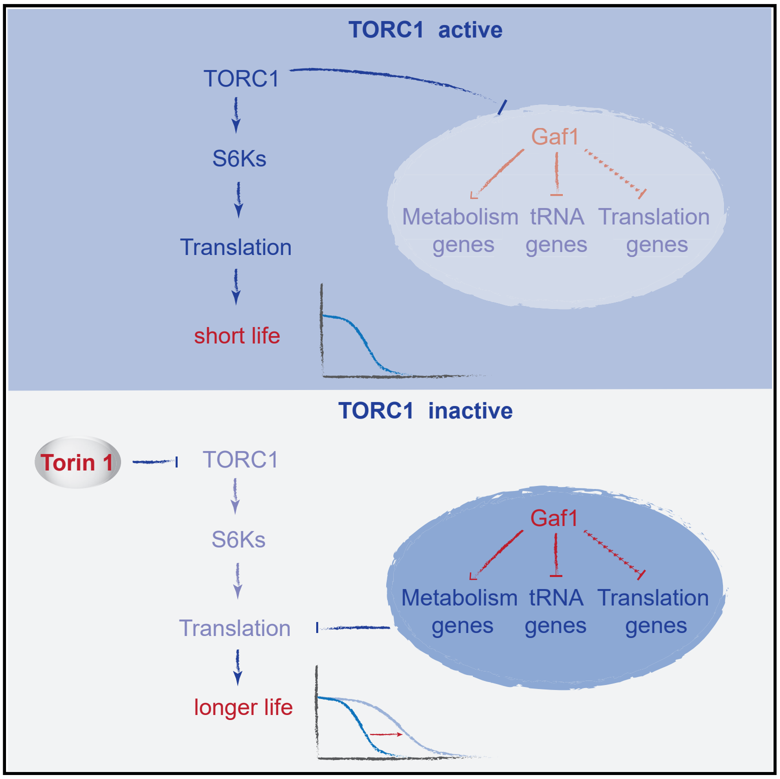

Highlights

- Cells lacking the GATA transcription factor Gaf1 grow normally after blocking TORC1
- Gaf1 is required for the normal chronological lifespan of non-dividing cells
- Gaf1 regulates transcription of protein-coding genes and of tRNA genes
- Upon TORC1 block, Gaf1 represses tRNAs to inhibit translation and promote longevity

Authors

María Rodríguez-López, Suam Gonzalez, Olivia Hillson, ..., Victor A. Tallada, Jürg Bähler, Charalampos Rallis

Correspondence

j.bahler@ucl.ac.uk (J.B.),
c.rallis@essex.ac.uk (C.R.)

In Brief

TORC1 signaling promotes protein translation and cellular aging. Rodríguez-López et al. show that this role of TORC1 is partly mediated via the transcription factor Gaf1. When TORC1 is blocked, Gaf1 extends the cellular lifespan by inhibiting the transcription not only of protein-coding genes but also of tRNA genes functioning in translation.

Rodríguez-López et al., 2020, Cell Reports *30*, 3240–3249
March 10, 2020 © 2020 The Author(s).
https://doi.org/10.1016/j.celrep.2020.02.058

CellPress

OPEN ACCESS
CellPress

Cell Reports
Report

The GATA Transcription Factor Gaf1 Represses tRNAs, Inhibits Growth, and Extends Chronological Lifespan Downstream of Fission Yeast TORC1

María Rodríguez-López,[1,7] Suam Gonzalez,[2,7] Olivia Hillson,[2,5] Edward Tunnacliffe,[1] Sandra Codlin,[1,6] Victor A. Tallada,[3] Jürg Bähler,[1,8,*] and Charalampos Rallis[1,2,4,*]
[1]Institute of Healthy Ageing and Department of Genetics, Evolution & Environment, University College London, London WC1E 6BT, UK
[2]School of Health, Sport and Bioscience, University of East London, Stratford Campus, London E14 4LZ, UK
[3]Centro Andaluz de Biología del Desarrollo, Universidad Pablo de Olavide/CSIC, 41013 Sevilla, Spain
[4]School of Life Sciences, University of Essex, Wivenhoe Park, Colchester CO4 3SQ, UK
[5]Present address: Department of Genetics, Evolution & Environment and Institute of Healthy Ageing, University College London, London WC1E 6BT, UK
[6]Present address: Department of Biological Sciences, Birkbeck College, University of London, London WC1E 7HX, UK
[7]These authors contributed equally
[8]Lead Contact
*Correspondence: j.bahler@ucl.ac.uk (J.B.), c.rallis@essex.ac.uk (C.R.)
https://doi.org/10.1016/j.celrep.2020.02.058

SUMMARY

Target of Rapamycin Complex 1 (TORC1) signaling promotes growth and aging. Inhibition of TORC1 leads to reduced protein translation, which promotes longevity. TORC1-dependent post-transcriptional regulation of protein translation has been well studied, while analogous transcriptional regulation is less understood. Here we screen fission yeast mutants for resistance to Torin1, which inhibits TORC1 and cell growth. Cells lacking the GATA factor Gaf1 (*gaf1Δ*) grow normally even in high doses of Torin1. The *gaf1Δ* mutation shortens the chronological lifespan of non-dividing cells and diminishes Torin1-mediated longevity. Expression profiling and genome-wide binding experiments show that upon TORC1 inhibition, Gaf1 directly upregulates genes for small-molecule metabolic pathways and indirectly represses genes for protein translation. Surprisingly, Gaf1 binds to and downregulates the tRNA genes, so it also functions as a transcription factor for RNA polymerase III. Thus, Gaf1 controls the transcription of both protein-coding and tRNA genes to inhibit translation and growth downstream of TORC1.

INTRODUCTION

The conserved Target of Rapamycin (TOR) signaling pathway is a key regulator for cellular growth and metabolism in response to nutrients and energy (Gonzalez and Rallis, 2017; González and Hall, 2017; Valvezan and Manning, 2019; Wei et al., 2013). TOR generally functions via two multi-protein complexes, TORC1 and TORC2, which coordinate distinct aspects of growth and associated processes (Hartmuth and Petersen, 2009; Ikai et al., 2011). TORC2 is not required for cell proliferation in fission yeast (*Schizosaccharomyces pombe*) but is required for sexual differentiation, stress response, and actin function (Matsuo et al., 2007; Weisman and Choder, 2001). TORC1 activates protein synthesis and other anabolic processes and inhibits autophagy and other catabolic processes. Active TORC1 functions on lysosomes, or vacuoles in yeast, in response to growth signals (Binda et al., 2009; Chia et al., 2017; Poüs and Codogno, 2011; Valbuena et al., 2012).

In all organisms tested, TORC1 promotes aging and shortens lifespan (Gonzalez and Rallis, 2017; González and Hall, 2017; Kaeberlein, 2010; Wei et al., 2013). Lifespan is influenced by multiple TORC1-dependent processes, including mitochondrial activity (Hill and Van Remmen, 2014), autophagy (Saxton and Sabatini, 2017), and protein translation (Bjedov and Partridge, 2011; Rallis et al., 2013). Protein translation is controlled post-transcriptionally by TORC1 via phosphorylation of ribosomal S6 kinase (S6K) and the translation factors eIF2α and 4E-BP (Ma and Blenis, 2009). Inhibition of S6K can extend lifespan in several organisms (Bjedov et al., 2010; Rallis et al., 2014; Roux et al., 2006; Selman et al., 2009).

Besides post-transcriptional mechanisms, TORC1 promotes translational capacity and aging via transcriptional regulation (Valvezan and Manning, 2019). It stimulates transcription of ribosomal RNAs via RNA polymerases I and III (RNA Pol I and RNA Pol III) (Iadevaia et al., 2014), although mechanisms are poorly understood. TORC1 may regulate RNA Pol I transcription via general transcription factors (Hannan et al., 2003; Mayer et al., 2004). TORC1 also regulates the conserved Maf1 factor, which inhibits RNA Pol III (Cai and Wei, 2015; Graczyk et al., 2018; Michels et al., 2010; Shor et al., 2010; Wei and Zheng, 2010; Wei et al., 2009). RNA Pol III transcribes the highly abundant 5S ribosomal RNAs and transfer RNAs (tRNAs), which are central for translation, besides other small RNAs (Arimbasseri and Maraia, 2016). Given the focus on protein-coding gene transcription, the regulation of RNA Pol III transcription is less well understood. A recent study shows that RNA Pol III activity limits the lifespan

 © 2020 The Author(s).
This is an open access article under the CC BY license (http://creativecommons.org/licenses/by/4.0/).

downstream of TORC1 (Filer et al., 2017). Altogether, these findings suggest that TORC1-mediated control of RNA Pol III transcription is universally important for translation and aging. However, no specific transcription factors have been identified that bind to RNA Pol III-dependent promoters and thus mediate translational control and lifespan.

The conserved *S. pombe* GATA transcription factor Gaf1 regulates responses to nitrogen limitation downstream of TORC1 (Laor et al., 2015). Gaf1 can regulate gene expression either positively or negatively (Kim et al., 2012). Here we show that Gaf1 is required for growth suppression upon TORC1 inhibition. Gaf1 binds not only to the promoters of certain protein-coding genes but also to the RNA Pol III-transcribed tRNA genes, which leads to their repression. Mutant cells lacking Gaf1 feature a shortened chronological lifespan. Our results uncover a transcription factor downstream of TORC1 that directly inhibits transcription of the tRNA genes, providing a mechanism for transcriptional control of global protein translation that prolongs lifespan.

RESULTS AND DISCUSSION

Genes Required for TOR-Mediated Growth Inhibition

TORC1 and TORC2 can be inhibited by Torin1, an ATP analog that blocks cell proliferation in *S. pombe* (Atkin et al., 2014; Thoreen et al., 2009). Using a low Torin1 dose (5 μM), *S. pombe* mutants have been screened for resistance and sensitivity to reduced TOR signaling (Lie et al., 2018). Here we screened mutants under a four-fold higher Torin1 dose (20 μM). This dose blocked cell growth (Figure 1A) and reduced the size of both cells and vacuoles (Figure 1B). Global protein translation was also reduced by Torin1, as reflected by reduced phosphorylation of ribosomal S6 protein and increased total and phosphorylated eIF2α (Figure 1C). Altogether, these phenotypes look like those triggered by caffeine and rapamycin that block TORC1 function (Rallis et al., 2013). We conclude that Torin1 leads to phenotypes that are diagnostic for TORC1 inhibition.

We screened for deletion mutants that can suppress the strong growth inhibition by 20 μM Torin1 (Figure 1D). Overall, 19 mutants were resistant to Torin1-mediated growth inhibition in all 4 repeats (Figure 1E; Table S1), 9 of which were identified in the previous screen (Lie et al., 2018). We independently validated these 19 mutants, both by PCR and by backcrossing to a wild-type strain. The backcrossed mutants were spotted on Torin1 plates to confirm linkage of the drug-resistant phenotype to the deletion cassette. Although wild-type cells did not grow in Torin1, all 19 mutants managed to grow to various extents in different concentrations of Torin1 (Figure 1F). Four mutants were resistant to Torin1 at all concentrations, showing similar growth as on untreated medium (Figure 1F, red frames).

Some mutants feature resistance to multiple rather than specific drugs (Dawson et al., 2008). To exclude this possibility for the Torin1-resistant mutants, we assayed their growth in four other drugs; this analysis showed that all mutants were at least as sensitive to the other drugs as the wild-type control (Figure S1A), indicating that their Torin1 resistance does not reflect multi-drug resistance. To exclude the possibility that resistance simply reflects that mutants cannot take up Torin1, we tested whether the Torin1-resistant mutants still showed other phenotypes of TORC1 inhibition (Figures 1B and 1C). The mutants still showed reduced ribosomal S6 protein phosphorylation after Torin1 treatment, except *aca1Δ* (Figure S1B), and decreased cell size (Figure S1C). These results indicate that Torin1 is taken up by the mutant cells, which differ in sensitivity to different TORC1 functions. Moreover, in all but the *aca1Δ* mutant, the growth resistance to Torin1 may be independent of translational control by ribosomal S6 phosphorylation.

The 19 genes identified in our screen function in limited cellular processes (Figure 1G; Table S1). Vesicular transport and vacuolar functions were associated with 13 genes, 6 of which encode components of endosomal sorting complexes required for transport (ESCRT). Many of these proteins are part of the Nbr1-mediated vacuolar targeting (NVT) autophagic system (Liu et al., 2015). The NVT pathway does not contain core Atg proteins but depends on ESCRTs and the multi-vesicular body to deliver soluble cargoes to the vacuole. How might vesicular transport and the NVT pathway relate to TOR signaling? Disruption of vesicle-mediated transport at the endosome triggers a metabolic signature similar to TORC1 inhibition (Mulleder et al., 2016). It is possible that TORC1 controls the NVT pathway or that some of our mutants affect TORC1 localization to the vacuole, thus rendering the system resistant to Torin1 inhibition. A gene from the screen encodes the GATA transcription factor Gaf1. In budding yeast, components of Golgi-to-vacuole trafficking are required for TORC1-responsive regulation of GATA factors (Fayyadkazan et al., 2014; Puria et al., 2008). Given our interest in TORC1-dependent gene regulation and the strong Torin1-resistance of *gaf1Δ* mutants (Figure 1G), we further analyzed the function of Gaf1.

Gaf1 Is Required for Normal Lifespan and Lifespan Extension by Torin1 Treatment

TORC1 inhibition through nutrient limitation or rapamycin prolongs chronological lifespan in *S. pombe* (Rallis et al., 2013, 2014), defined as the time post-mitotic cells remain viable in stationary phase. Given that Gaf1 is required to arrest growth upon TOR inhibition, we hypothesized that Gaf1 may also play a role in chronological lifespan. Indeed, *gaf1Δ* cells were shorter lived, with median and maximum lifespans of 3 and 16 days, respectively, compared with 5 and 20 days for wild-type cells (Figure 2). Thus, Gaf1 is required for the normal lifespan of non-dividing cells.

Torin1 increases lifespan in flies (Mason et al., 2018) and suppresses senescence in human tissue cultures (Leontieva and Blagosklonny, 2016). To analyze the effect of Torin1 on chronological lifespan in *S. pombe*, and any role of Gaf1 in this condition, we pre-treated exponentially growing wild-type and *gaf1Δ* cells with Torin1 and tested for subsequent effects on lifespan during the stationary phase. Torin1 substantially prolonged lifespan in wild-type cells, with median and maximum lifespans of 18 and 33 days, respectively, compared with 5 and 20 days in untreated cells (Figure 2A). In *gaf1Δ* cells, Torin1 also prolonged lifespan but to a lesser extent than in wild-type cells, with median and maximum lifespans of ~13 and 30 days, respectively (Figure 2A). To quantify the role of Gaf1 in Torin1-mediated longevity, we calculated the areas under the curve (AUCs, measured as days × percentage of survival from lifespan assays). In

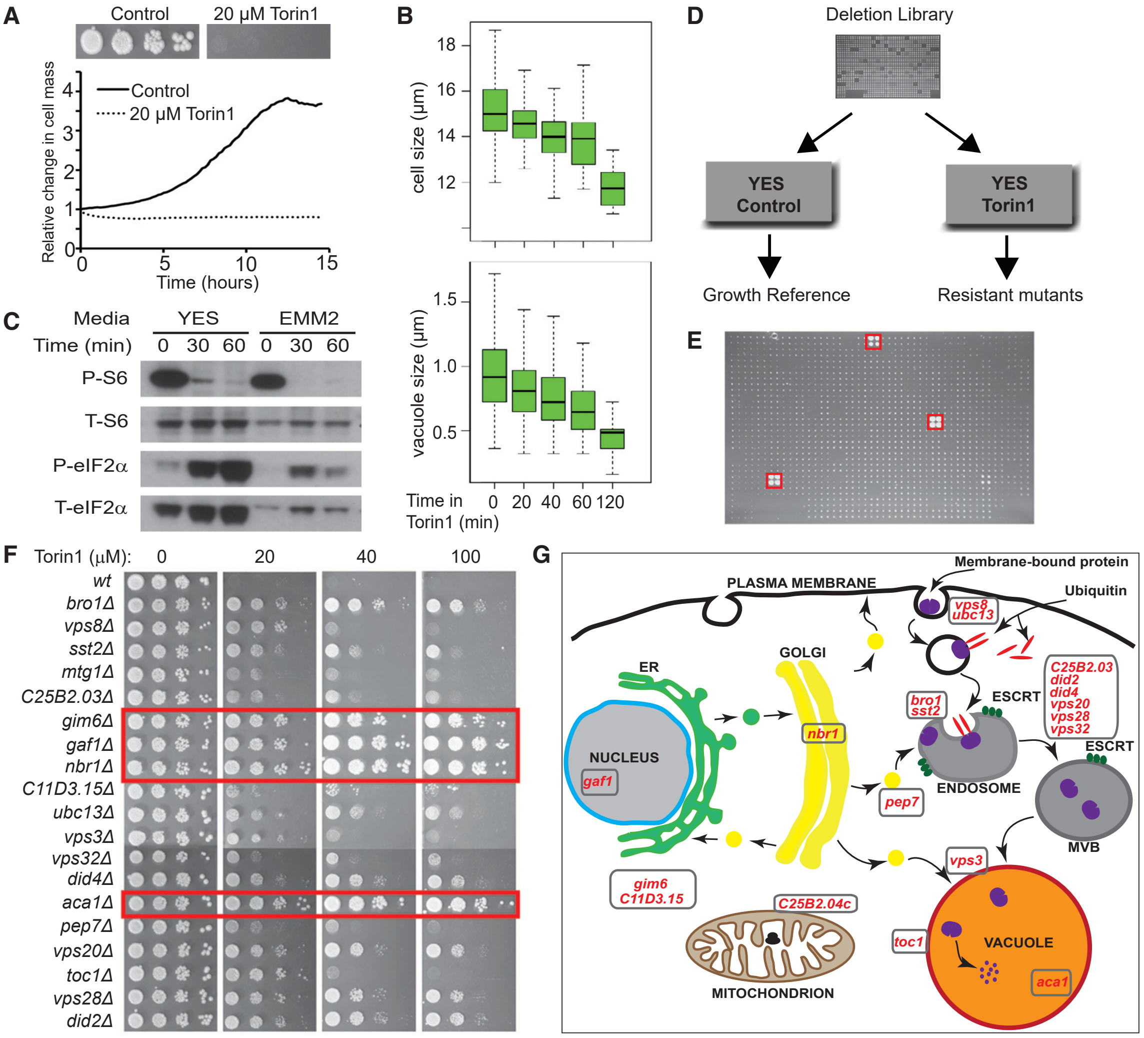

Figure 1. Screen for Torin1-Resistant Mutants

(A) Torin1 blocks cell proliferation. Top: ten-fold serial dilutions of wild-type (WT) cells spotted on rich solid medium. Bottom: growth profiles in rich liquid medium using a microfermentor, in the absence (control) and presence of Torin1.

(B) Torin1 leads to decreased cell and vacuole sizes. Sizes of septated WT cells (top) and vacuoles (bottom) during Torin1 treatment.

(C) Torin1 alters phosphorylation status of translational regulators. Phosphorylated (P) and total amounts (T) of ribosomal S6 and eIF2α proteins in WT cells following Torin1 treatment in rich (YES) or minimal (EMM2) media.

(D) Design of genome-wide screens to identify mutants resistant to Torin1-mediated growth inhibition. We screened Bioneer version 2 (3,005 mutants) and Bioneer version 5 (3,420 mutants) of deletion libraries (Kim et al., 2010) in two independent repeats each, using 20 µM Torin1 on rich solid medium (YES).

(E) Example of deletion library plate with Torin1, containing 1,536 colonies with each mutant printed in quadruplicate. Red boxes indicate three Torin1-resistant mutants.

(F) Torin1 sensitivity test using spotting assays for a WT control and the 19 resistant mutants identified, using different Torin1 concentrations as indicated. Red frames: 4 mutants showing strong resistance to all Torin1 concentrations tested.

(G) Cellular processes associated with the 19 genes (red) required for Torin1-mediated growth inhibition.

See also Figure S1 and Table S1.

wild-type cells, the lifespan was prolonged from an average AUC of 1,044 to 2,689 (increase of 1,645), whereas in *gaf1Δ* cells, the lifespan was prolonged to a lesser extent, from an average AUC of 681 to 1,709 (increase of 1,027) (Figure 2B). We conclude that Gaf1 is also required for the full lifespan extension resulting from Torin1-mediated TOR inhibition during cell proliferation. However, Torin1 still can prolong lifespan considerably without Gaf1, indicating that other factors contribute to this longevity. Indeed, we have identified several proteins required for lifespan extension when TORC1 is inhibited, including the S6K protein Sck2 (Rallis et al., 2014).

Gaf1-Dependent Transcriptome Regulation following TOR Inhibition

Given that Gaf1 was essential for growth inhibition by Torin1 (Figure 1G), we further analyzed its function in this condition. Gaf1

OPEN
ACCESS
CellPress

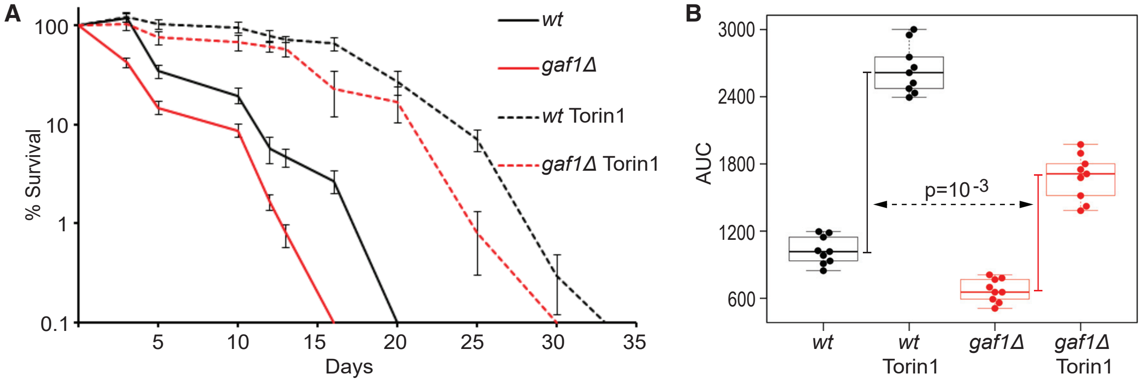

Figure 2. Gaf1 Is Required for Longevity

(A) Chronological lifespan assays in WT and *gaf1Δ* cells grown in EMM2 in the absence or presence of 8 μM Torin1. Error bars represent SDs from 3 independent cell cultures, each measured 3 times per time point.

(B) AUC for lifespan assays of WT and *gaf1Δ* mutant cells without or with Torin1 treatment. Vertical bars: Torin1-mediated increase in average AUC values for WT (black) and *gaf1Δ* (red), with the p value reflecting the significantly larger lifespan increase in WT than in *gaf1Δ* cells.

accumulated in the nucleus within a few minutes following treatment with Torin1 (Figure 3A) or with caffeine and rapamycin (Figure S2), drugs that inhibit TORC1, but not TORC2 (Rallis et al., 2013). Consistently, Gaf1 is known to translocate to the nucleus during nitrogen limitation, which also inhibits TORC1, and biochemical analyses have shown that Gaf1 localization and phosphorylation depend on TORC1 activity (Laor et al., 2015; Ma et al., 2015). This regulation of GATA transcription factors is conserved: budding yeast Gln3 and Gat1 (Broach, 2012) and mammalian GATA6 (Xie et al., 2015) are also sequestered in the cytoplasm by active TORC1 and translocate to the nucleus upon TORC1 inhibition.

In *S. pombe*, Gaf1 activates genes functioning in amino-acid transport but represses *ste11*, encoding a master regulator for meiotic differentiation (Kim et al., 2012; Ma et al., 2015). To systematically identify Gaf1-dependent transcripts, we performed microarray analyses of wild-type and *gaf1Δ* cells, both before and after Torin1 treatment. Before Torin1 treatment, wild-type and *gaf1Δ* cells showed similar expression signatures (Figures 3B and 3C). We conclude that in proliferating cells, Gaf1 plays no or a negligible role in gene regulation, consistent with its cytoplasmic localization when TORC1 is active (Figure 3A; Laor et al., 2015).

However, Torin1 treatment resulted in substantial transcriptome changes in both wild-type and *gaf1Δ* cells, but in *gaf1Δ* cells, the expression signature markedly differed from the signature in wild-type cells (Figures 3B and 3C). Overall, 90 and 108 genes consistently showed $\geq$1.5-fold higher or lower expression, respectively, in *gaf1Δ* relative to wild-type cells after Torin1 treatment (Figure 3B; Table S2). Cells treated with caffeine and rapamycin, which inhibit TORC1, but not TORC2 (Rallis et al., 2013), showed similar expression signatures as Torin1-treated cells in both wild-type and *gaf1Δ* cells (Figures 3B and 3C). This result indicates that the Torin1-mediated expression signatures in wild-type and *gaf1Δ* cells reflect TORC1 inhibition. We conclude that after TORC1 inhibition, Gaf1 affects the expression of $\sim$200 genes, either positively or negatively.

We performed functional enrichment analyses for these Gaf1-dependent genes using AnGeLi and g:profiler (Bitton et al., 2015; Raudvere et al., 2019). The 90 genes that were expressed higher in *gaf1Δ* than in wild-type cells (i.e., genes repressed by Gaf1) were typically downregulated in Torin1-treated wild-type cells but less so in *gaf1Δ* cells (Figure 3B). These genes were enriched in anabolic processes such as biosynthesis (61 genes, $p = 9.4 \times 10^{-10}$), ribosome biogenesis (19 genes, $p = 1.6 \times 10^{-3}$), and cytoplasmic translation (31 genes, $p = 1.0 \times 10^{-16}$), including 25 genes encoding ribosomal proteins. In budding yeast and worms, genetic inhibition of ribosomal proteins leads to lifespan extension (Hansen et al., 2008; McCormick et al., 2015). Figure S3 visualizes all Gene Ontology (GO) biological processes enriched among the 90 genes. Many of these genes are also repressed as part of the core environmental stress response (43 genes, $p = 1.4 \times 10^{-20}$; Chen et al., 2003) and are highly expressed in proliferating cells (mean of 46.9 mRNA copies/cell versus 7.5 copies for all mRNAs, $p = 1.2 \times 10^{-26}$; Marguerat et al., 2012). We conclude that upon TORC1 inhibition, Gaf1 contributes to the downregulation of highly expressed genes functioning in protein synthesis.

The 108 genes that were expressed lower in *gaf1Δ* than in wild-type cells (i.e., genes induced by Gaf1) were typically upregulated in Torin1-treated wild-type cells but less so in *gaf1Δ* cells (Figure 3B). These genes were enriched in several metabolic processes of small molecules, including organonitrogen compounds (43 genes, $p = 4.6 \times 10^{-14}$), amino acids (18 genes, $p = 4.1 \times 10^{-5}$), urea (6 genes, $p = 7.3 \times 10^{-5}$), and organic acids (20 genes, $p = 0.001$) (Figure S3). There was also a substantial overlap with genes that are induced by nitrogen limitation (43 genes, $p = 1.3 \times 10^{-29}$; Mata et al., 2002) and genes that are periodically expressed during the cell cycle (41 genes, $p = 1.6 \times 10^{-12}$; Marguerat et al., 2006), including 9 histone genes. These results suggest a Gaf1-dependent transcriptional program to adjust the metabolism of amino acids and other molecules, possibly to recycle nutrients under conditions that do not allow rapid proliferation. Similar gene-expression changes are mediated by budding yeast Gln3 and Gat1 under conditions of TORC1 inhibition (Kuroda et al., 2019; Scherens et al., 2006). Altogether, these findings indicate that Gaf1 regulates

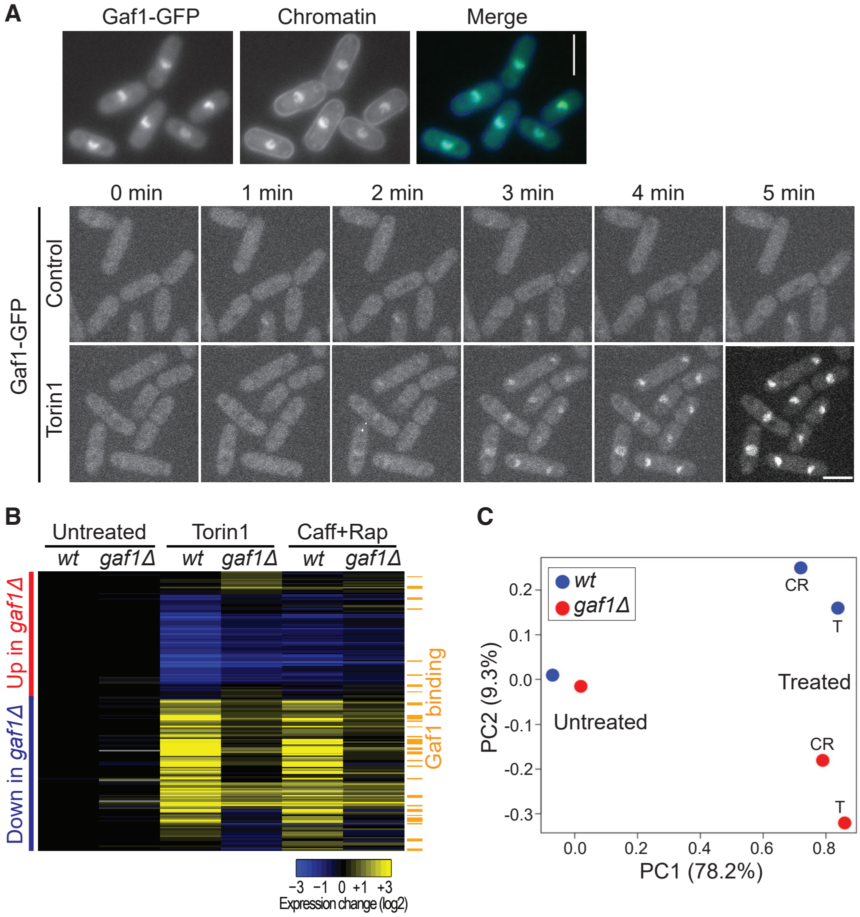

Figure 3. Gaf1-Dependent Gene Expression

(A) Top panels: fluorescence microscopy of cells expressing GFP-tagged Gaf1 (left) with chromatin stained by Hoechst 33342 (middle) after 10 min of exposure to 20 μM Torin1. Bottom panels: fluorescence microscopy of live Gaf1-GFP cells, showing stack projections of 1-min time lapses in rich medium. Cells are shown before (0 min) and in 1-min intervals after addition of either DMSO (solvent control; upper panels) or Torin1 (20 μM final; lower panels). Gaf1-GFP is visible inside the nucleus within 3 min after Torin1 addition. Scale bars: 5 μm. See also Figure S2.

(B) Hierarchical clustering of microarray data. Columns represent WT or *gaf1* mutants (*gaf1Δ*) before (untreated) and after 1 h of treatment with 20 μM Torin1 or with 10 mM caffeine and 100 ng/mL of rapamycin (Caff+Rap). Rows represent the 198 genes whose mRNA levels changed ≥1.5-fold in Torin1-treated *gaf1Δ* cells relative to WT cells, consisting of 90 genes showing higher expression (red bar) and 108 genes showing lower expression (blue bar) in *gaf1Δ* cells. In untreated cells, only 3 genes showed ≥1.5-fold expression changes in *gaf1Δ* relative to WT cells. Average RNA expression changes (from 2 independent repeats) in the different genetic and pharmacological conditions relative to WT control cells are color coded as shown. The orange bars indicate 43 genes whose promoters were bound by Gaf1 after 60 min with Torin1. See also Figure S3.

(C) Principal-component (PC) analysis of all genes measured by microarrays. PC1 separates untreated cells from cells treated with Torin1 (T) or caffeine and rapamycin (CT), while PC2 separates WT (blue) from *gaf1* mutants (*gaf1Δ*, red). Percentages of the x and y axes show the contribution of the corresponding PC to the difference in the data.

physiological changes supporting the growth arrest triggered by TORC1 inhibition.

Gaf1 Binds to Both Coding and tRNA Genes following TOR Inhibition

The microarray analyses identified genes whose expression depends on Gaf1, some of which may be directly regulated by Gaf1. To detect gene promoters bound by Gaf1, we performed chromatin immunoprecipitation sequencing (ChIP-seq) of Gaf1-GFP cells. The number of Gaf1-bound promoters increased from 165 before Torin1 treatment to 454 after Torin1 treatment, with 93 genes in common between the two conditions (Figure 4A). Gaf1 binding sites upstream of close, divergently expressed genes were assigned to both genes. The Gaf1 target genes after Torin1 treatment consisted of 245 protein-coding genes and 209 non-coding genes (Table S3).

The protein-coding Gaf1 target genes were significantly enriched in metabolic processes of organonitrogen compounds (55 genes, $p = 1.4 \times 10^{-6}$), including nucleotides (24 genes, $p = 0.0009$) and organic acids (34 genes, $p = 0.0003$) (Figure S4). They were also enriched for genes induced by nitrogen limitation (40 genes, $p = 1.6 \times 10^{-11}$) and periodically expressed during the cell cycle (53 genes, $p = 4.5 \times 10^{-6}$). Overall, these target genes showed similar functional enrichments to the genes whose expression was induced by Gaf1. Accordingly, Gaf1 binding sites were enriched among the genes induced by Gaf1 (Figure 3B, orange bars). Moreover, most protein-coding genes bound by Gaf1 were induced by TORC1 inhibition but less so in *gaf1Δ* cells, leading to distinct clusters for wild-type and mutant conditions (Figure 4B). We conclude that coding Gaf1 target genes are mostly upregulated by Gaf1 when TORC1 is inhibited.

Notably, Gaf1 bound to the promoters of 20 transcription factor genes (Table S3; Figure S4). Typically, these factors were induced in wild-type cells after TORC1 inhibition but less so in *gaf1Δ* cells. Many of these factors are involved in stress responses or cell-cycle regulation, including Atf1 (Wilkinson et al., 1996), Cbf12 (Chen et al., 2003), Fep1 (Bekker et al., 1991), Fil1 (Duncan et al., 2018), Hsr1 (Chen et al., 2008), Klf1 (Shimanuki et al., 2013), Loz1 (Corkins et al., 2013), Pap1 (Chen et al., 2008), Php3 (Mercier et al., 2006), and Sep1 (Rustici et al., 2004). These transcription-factor targets indicate that Gaf1 may indirectly control some Gaf1-dependent genes via other transcription factors. Gaf1 inhibited the expression of many genes functioning in translation (Figure S3), but these genes were not among its direct binding targets. These genes may thus be indirectly regulated by Gaf1; for example, Atf1 represses

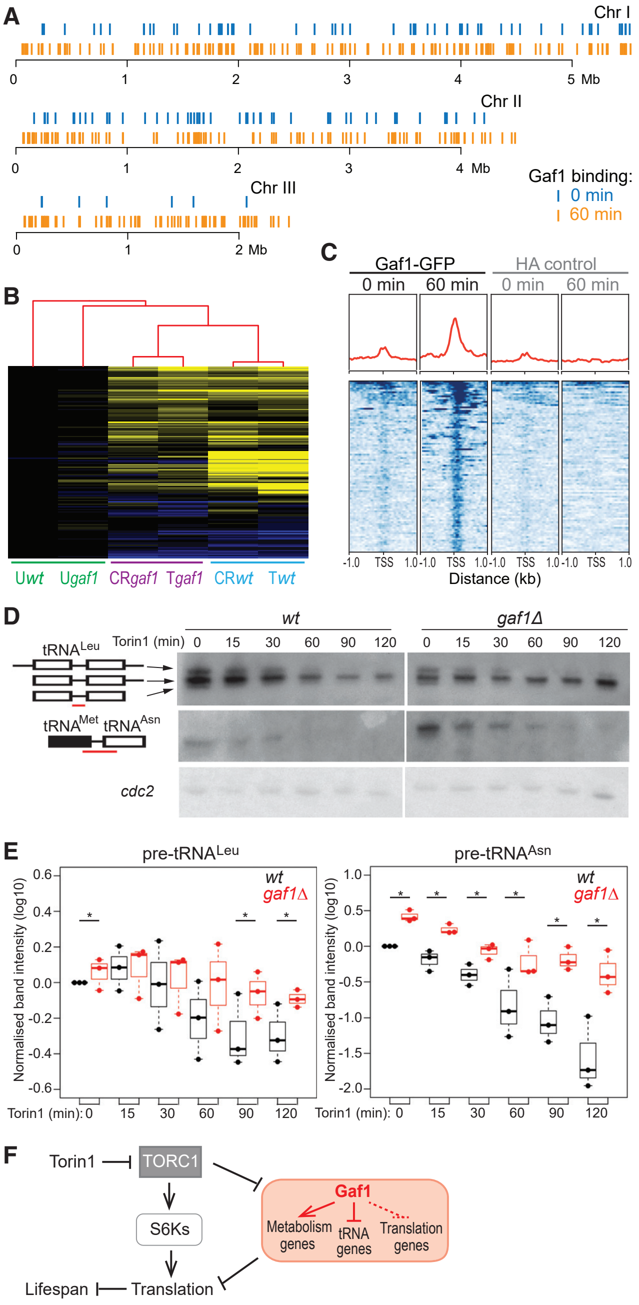

Figure 4. Gaf1 Regulation of Protein-Coding and tRNA Genes

(A) Gaf1 binding sites across the 3 *S. pombe* chromosomes, before (0 min, blue) and after (60 min, orange) treatment with 20 μM Torin1. See also Figure S4.

(B) Hierarchical clustering of microarray data for 150 protein-coding genes bound by Gaf1 after Torin1 treatment and for which expression data were available for all conditions. The conditions have been clustered as well (red tree on top) and are grouped as follows: untreated WT and *gaf1Δ* cells (U*wt*, U*gaf1*), caffeine+rapamycin- or Torin1-treated *gaf1Δ* cells (CR*gaf1*, T*gaf1*), and caffeine+rapamycin- or Torin1-treated WT cells (CR*wt*, T*wt*). Expression changes are color coded as in Figure 3B.

(C) Gaf1 shows increased binding to tRNA genes after Torin1 treatment. Top, red curves: average Gaf1 binding profiles aligned to transcription start sites (TSSs) of all *S. pombe* tRNA genes before (0 min) and after (60 min) Torin1 treatment, along with corresponding control ChIP-seq data (hemagglutinin [HA]). Bottom: heatmaps of Gaf1 binding around the TSS of all 196 tRNAs, ordered by normalized ChIP-seq coverage. See also Figure S5.

(D) Northern blots of precursor tRNAs for leucine (top) and asparagine (middle) from WT and *gaf1Δ* cells, treated with 20 μM Torin1 over 120 min as indicated. Probes to detect precursor tRNAs, indicated with red bars, are as described before (Otsubo et al., 2018). Probes for *cdc2* were used as loading control (bottom).

(E) Northern quantitation of leucine and asparagine precursor tRNAs relative to WT time 0 and normalized to loading controls (three independent repeats shown as dots). Asterisks denote significant differences in pre-tRNA levels between WT and *gaf1Δ* cells from same time point (t test, $p < 0.05$).

(F) Model for Gaf1-mediated transcriptional repression of translation downstream of TORC1. Following TORC1 inhibition, Gaf1 activates the transcription of genes for small-molecule metabolic pathways and represses the transcription of tRNAs and other genes functioning in translation (the latter via indirect control, hatched). Together with the S6K-mediated translational control (Ma and Blenis, 2009), this transcriptional branch contributes to longevity.

translation-related genes during stress (Chen et al., 2008), raising the possibility that it also represses these genes during TORC1 inhibition in a Gaf1-dependent manner.

The 209 non-coding genes among the Gaf1 targets included 82 tRNA genes and a small nucleolar RNA (snoRNA) involved in tRNA regulation, besides large non-coding RNAs (Table S3). Coverage plotting indicated that Gaf1 binds to all tRNA genes that are clustered in *S. pombe* (Figure 4C; Figure S5). Binding occurred near the transcription start sites for tRNA genes and strongly increased after Torin1 treatment (Figure 4C). We conclude that Gaf1 binds not only to genes transcribed by RNA Pol II but also to the tRNA genes transcribed by RNA Pol III. To address whether Gaf1 represses or activates tRNAs, we performed northern analyses of tRNA gene expression as a function of Gaf1 and Torin1. The abundant mature tRNAs are rapidly processed from precursor tRNAs, which need to be assayed to detect expression changes (Otsubo et al., 2018). The expression of tRNA precursors decreased during Torin1 treatment in wild-type cells, while in *gaf1Δ* cells their expression was higher and showed a delayed and less pronounced decrease, especially at later time points (Figures 4D and 4E). We conclude that Gaf1 binds to tRNA genes and inhibits their expression upon TOR inhibition.

Downregulation of precursor tRNA expression is required for TORC1 inhibition in *S. pombe* (Otsubo et al., 2018), indicating that tRNAs can act upstream of TORC1. Our experiments, conversely, point to a mechanism of tRNA regulation downstream of TORC1. Altogether, these findings suggest regulatory feedback, involving precursor tRNAs, TORC1, and Gaf1, to match tRNA expression to physiological requirements. Our results reveal a transcription factor that not only controls RNA Pol II-mediated expression of genes functioning in translation- and metabolism-related processes but also globally inhibits RNA Pol III-mediated expression of tRNAs. It will be interesting to test whether the latter function is conserved for orthologous GATA transcription factors. Studies of Gln3 and Gat1 function in budding yeast have excluded tRNAs and are therefore not conclusive with respect to tRNA gene regulation (Kuroda et al., 2019; Scherens et al., 2006).

Conclusions

The GATA transcription factor Gaf1 is essential for blocking cell proliferation with Torin1; in its absence, cell growth remains normal, even in high doses of Torin1 (Figure 1G). Gaf1 is also required for normal chronological lifespan and contributes to, but is not necessary for, the longevity of Torin1-treated cells (Figure 2). Upon TORC1 inhibition, Gaf1 inhibits the expression of genes functioning in protein translation, including protein-coding genes, which may be indirectly controlled by Gaf1, and tRNA genes, which are binding targets of Gaf1 (Figures 3 and 4). Gaf1 also positively controls genes functioning in metabolic pathways for nitrogen-containing molecules, which support the adaptation to lowered protein synthesis. Thus, Gaf1 can directly regulate both RNA Pol II- and RNA Pol III-transcribed genes. It is possible that Gaf1 elicits its repressor activity at tRNA genes by recruiting a histone deacetylase: work in *S. pombe* has identified potential loading sites for Clr6 complex components at tRNAs (Zilio et al., 2014). Downregulation of global protein translation is beneficial for longevity in all organisms studied, including *S. pombe* (Kaeberlein and Kennedy, 2011; Rallis and Bähler, 2013). Given its role in repressing diverse translation-related factors, Gaf1 may inhibit aging by contributing to the downregulation of translation upon TORC1 inhibition (Figure 4F). Gaf1 thus defines a transcription-based branch of translational and metabolic control downstream of TORC1, in parallel to the post-translational branch exerted by translational regulators like S6K (Figure 4F). This transcriptional branch is essential for growth inhibition triggered by lowered TORC1 activity.

Repression of RNA Pol III prolongs lifespan in yeast, worms, and flies and is required for the lifespan extension mediated by TORC1 inhibition (Filer et al., 2017). Besides general transcription factors such as TFIIIB, TFIIIC, and TBP, several factors control RNA Pol III transcription without directly binding to DNA (Hummel et al., 2019), including the RNA Pol III inhibitor Maf1, the coactivator PNRC, and MYC, which interacts with the RNA Pol III basal apparatus (Campbell and White, 2014; Graczyk et al., 2018; Zhou et al., 2007). To our knowledge, the TORC1 target Gaf1 is the first specific transcription factor shown to globally bind to and repress the tRNA genes. Thus, Gaf1 could exert the aging-associated function of RNA Pol III. Recent work in flies shows that GATA transcription factors can mediate the effects of dietary restriction on lifespan (Dobson et al., 2018). This finding raises the possibility that Gaf1 regulation of aging-related processes is conserved and that other GATA factors exert similar functions downstream of TORC1. The mouse and human ortholog of Gaf1, GATA6, is involved in differentiation, stem cell maintenance, and cancer (Viger et al., 2008; Wamaitha et al., 2015; Zhong et al., 2011). It is plausible that GATA6 exerts these important functions by regulating translation-related genes, including tRNAs.

STAR★METHODS

Detailed methods are provided in the online version of this paper and include the following:

- KEY RESOURCES TABLE
- LEAD CONTACT AND MATERIALS AVAILABILITY
- EXPERIMENTAL MODEL AND SUBJECT DETAILS
- METHOD DETAILS
  - Drug sensitivity assays
  - Measurement of cell size and fluorescence microscopy
  - Measurement of vacuolar size
  - Chronological lifespan assay
  - High-throughput genetic screening
  - Growth assay
  - Western blotting and antibodies
  - Microarrays
  - ChIP-seq
  - Northern analyses
- QUANTIFICATION AND STATISTICAL ANALYSIS
- DATA AND CODE AVAILABILITY
  - Scripts for Analysis
  - Dataset Hosting

SUPPLEMENTAL INFORMATION

Supplemental Information can be found online at https://doi.org/10.1016/j.celrep.2020.02.058.

ACKNOWLEDGMENTS

We thank Nazif Alic for critical reading of the manuscript and Pawan Dhami (Genomics and Genome Engineering Facility, funded by the Cancer Research UK-UCL Centre) for help with sequencing. This research was funded by a Wellcome Trust Senior Investigator Award grant 095598/Z/11/Z and BBSRC project grant BB/R009597/1 (to J.B.) and QR funds and a UEL back-to-the-bench project grant (to C.R.). S.G. was funded by a competitive UEL PhD studentship grant awarded to C.R.

AUTHOR CONTRIBUTIONS

Conceptualization, C.R.; Methodology, C.R., S.G., M.R.-L., V.A.T., and S.C.; Investigation, C.R., M.R.-L., S.G., O.H., V.A.T., E.T., S.C., and J.B.; Formal Analysis, C.R., M.R.-L., S.G., and J.B.; Writing, C.R. and J.B.; Funding Acquisition, C.R. and J.B.; Supervision, C.R. and J.B.

DECLARATION OF INTERESTS

The authors declare no competing interests.

Received: August 6, 2019
Revised: December 17, 2019
Accepted: February 13, 2020
Published: March 10, 2020

REFERENCES

Arimbasseri, A.G., and Maraia, R.J. (2016). RNA polymerase III advances: structural and tRNA functional views. Trends Biochem. Sci. *41*, 546–559.

Atkin, J., Halova, L., Ferguson, J., Hitchin, J.R., Lichawska-Cieslar, A., Jordan, A.M., Pines, J., Wellbrock, C., and Petersen, J. (2014). Torin1-mediated TOR kinase inhibition reduces Wee1 levels and advances mitotic commitment in fission yeast and HeLa cells. J. Cell Sci. *127*, 1346–1356.

Bahler, J., Wu, J.Q., Longtine, M.S., Shah, N.G., McKenzie, A., 3rd, Steever, A.B., Wach, A., Philippsen, P., and Pringle, J.R. (1998). Heterologous modules for efficient and versatile PCR-based gene targeting in Schizosaccharomyces pombe. Yeast *14*, 943–951.

Bekker, A., Von Hagen, S., and Yarmush, J. (1991). A Macintosh Hypercard stack to simulate the pharmacokinetics of infusion of intravenous anesthetic drugs (NarSim). Comput. Appl. Biosci. *7*, 531–532.

Binda, M., Péli-Gulli, M.P., Bonfils, G., Panchaud, N., Urban, J., Sturgill, T.W., Loewith, R., and De Virgilio, C. (2009). The Vam6 GEF controls TORC1 by activating the EGO complex. Mol. Cell *35*, 563–573.

Bitton, D.A., Schubert, F., Dey, S., Okoniewski, M., Smith, G.C., Khadayate, S., Pancaldi, V., Wood, V., and Bähler, J. (2015). AnGeLi: a tool for the analysis of gene lists from fission yeast. Front. Genet. *6*, 330.

Bjedov, I., and Partridge, L. (2011). A longer and healthier life with TOR down-regulation: genetics and drugs. Biochem. Soc. Trans. *39*, 460–465.

Bjedov, I., Toivonen, J.M., Kerr, F., Slack, C., Jacobson, J., Foley, A., and Partridge, L. (2010). Mechanisms of life span extension by rapamycin in the fruit fly *Drosophila melanogaster*. Cell Metab. *11*, 35–46.

Broach, J.R. (2012). Nutritional control of growth and development in yeast. Genetics *192*, 73–105.

Cai, Y., and Wei, Y.H. (2015). Distinct regulation of Maf1 for lifespan extension by Protein kinase A and Sch9. Aging (Albany N.Y.) *7*, 133–143.

Campbell, K.J., and White, R.J. (2014). MYC regulation of cell growth through control of transcription by RNA polymerases I and III. Cold Spring Harb. Perspect. Med. *4*, a018408.

Chen, D., Toone, W.M., Mata, J., Lyne, R., Burns, G., Kivinen, K., Brazma, A., Jones, N., and Bähler, J. (2003). Global transcriptional responses of fission yeast to environmental stress. Mol. Biol. Cell *14*, 214–229.

Chen, D., Wilkinson, C.R.M., Watt, S., Penkett, C.J., Toone, W.M., Jones, N., and Bähler, J. (2008). Multiple pathways differentially regulate global oxidative stress responses in fission yeast. Mol. Biol. Cell *19*, 308–317.

Chia, K.H., Fukuda, T., Sofyantoro, F., Matsuda, T., Amai, T., and Shiozaki, K. (2017). Ragulator and GATOR1 complexes promote fission yeast growth by attenuating TOR complex 1 through Rag GTPases. eLife *6*, e30880.

Clément-Ziza, M., Marsellach, F.X., Codlin, S., Papadakis, M.A., Reinhardt, S., Rodríguez-López, M., Martin, S., Marguerat, S., Schmidt, A., Lee, E., et al. (2014). Natural genetic variation impacts expression levels of coding, non-coding, and antisense transcripts in fission yeast. Mol. Syst. Biol. *10*, 764.

Codlin, S., and Mole, S.E. (2009). *S. pombe btn1*, the orthologue of the Batten disease gene *CLN3*, is required for vacuole protein sorting of Cpy1p and Golgi exit of Vps10p. J. Cell Sci. *122*, 1163–1173.

Corkins, M.E., May, M., Ehrensberger, K.M., Hu, Y.M., Liu, Y.H., Bloor, S.D., Jenkins, B., Runge, K.W., and Bird, A.J. (2013). Zinc finger protein Loz1 is required for zinc-responsive regulation of gene expression in fission yeast. Proc. Natl. Acad. Sci. USA *110*, 15371–15376.

Dawson, K., Toone, W.M., Jones, N., and Wilkinson, C.R.M. (2008). Loss of regulators of vacuolar ATPase function and ceramide synthesis results in multidrug sensitivity in *Schizosaccharomyces pombe*. Eukaryot. Cell *7*, 926–937.

Dobson, A.J., He, X., Blanc, E., Bolukbasi, E., Feseha, Y., Yang, M., and Piper, M.D.W. (2018). Tissue-specific transcriptome profiling of *Drosophila* reveals roles for GATA transcription factors in longevity by dietary restriction. NPJ Aging Mech. Dis. *4*, 5.

Duncan, C.D.S., Rodríguez-López, M., Ruis, P., Bähler, J., and Mata, J. (2018). General amino acid control in fission yeast is regulated by a nonconserved transcription factor, with functions analogous to Gcn4/Atf4. Proc. Natl. Acad. Sci. USA *115*, E1829–E1838.

Fayyadkazan, M., Tate, J.J., Vierendeels, F., Cooper, T.G., Dubois, E., and Georis, I. (2014). Components of Golgi-to-vacuole trafficking are required for nitrogen- and TORC1-responsive regulation of the yeast GATA factors. MicrobiologyOpen *3*, 271–287.

Filer, D., Thompson, M.A., Takhaveev, V., Dobson, A.J., Kotronaki, I., Green, J.W.M., Heinemann, M., Tullet, J.M.A., and Alic, N. (2017). RNA polymerase III limits longevity downstream of TORC1. Nature *552*, 263–267.

González, A., and Hall, M.N. (2017). Nutrient sensing and TOR signaling in yeast and mammals. EMBO J. *36*, 397–408.

Gonzalez, S., and Rallis, C. (2017). The TOR signaling pathway in spatial and temporal control of cell size and growth. Front. Cell Dev. Biol. *5*, 61.

Graczyk, D., Cieśla, M., and Boguta, M. (2018). Regulation of tRNA synthesis by the general transcription factors of RNA polymerase III—TFIIIB and TFIIIC, and by the MAF1 protein. Biochim. Biophys. Acta. Gene Regul. Mech. *1861*, 320–329.

Guo, Y., Mahony, S., and Gifford, D.K. (2012). High resolution genome wide binding event finding and motif discovery reveals transcription factor spatial binding constraints. PLoS Comput. Biol. *8*, e1002638.

Hannan, K.M., Brandenburger, Y., Jenkins, A., Sharkey, K., Cavanaugh, A., Rothblum, L., Moss, T., Poortinga, G., McArthur, G.A., Pearson, R.B., and Hannan, R.D. (2003). mTOR-dependent regulation of ribosomal gene transcription requires S6K1 and is mediated by phosphorylation of the carboxy-terminal activation domain of the nucleolar transcription factor UBF. Mol. Cell. Biol. *23*, 8862–8877.

Hansen, M., Chandra, A., Mitic, L.L., Onken, B., Driscoll, M., and Kenyon, C. (2008). A role for autophagy in the extension of lifespan by dietary restriction in *C. elegans*. PLoS Genet. *4*, e24.

Hartmuth, S., and Petersen, J. (2009). Fission yeast Tor1 functions as part of TORC1 to control mitotic entry through the stress MAPK pathway following nutrient stress. J. Cell Sci. *122*, 1737–1746.

Hill, S., and Van Remmen, H. (2014). Mitochondrial stress signaling in longevity: a new role for mitochondrial function in aging. Redox Biol. *2*, 936–944.

Hummel, G., Warren, J., and Drouard, L. (2019). The multi-faceted regulation of nuclear tRNA gene transcription. IUBMB Life *71*, 1099–1108.

Iadevaia, V., Liu, R., and Proud, C.G. (2014). mTORC1 signaling controls multiple steps in ribosome biogenesis. Semin. Cell Dev. Biol. *36*, 113–120.

Ikai, N., Nakazawa, N., Hayashi, T., and Yanagida, M. (2011). The reverse, but coordinated, roles of Tor2 (TORC1) and Tor1 (TORC2) kinases for growth, cell cycle and separase-mediated mitosis in *Schizosaccharomyces pombe*. Open Biol. *1*, 110007.

Kaeberlein, M. (2010). Lessons on longevity from budding yeast. Nature *464*, 513–519.

Kaeberlein, M., and Kennedy, B.K. (2011). Hot topics in aging research: protein translation and TOR signaling, 2010. Aging Cell *10*, 185–190.

Kahm, M., Hasenbrink, G., Lichtenberg-Fraté, H., Ludwig, J., and Kschischo, M. (2010). grofit: Fitting biological growth curves with R. J. Stat. Soft. *33*, 1–21.

Kim, D.U., Hayles, J., Kim, D., Wood, V., Park, H.O., Won, M., Yoo, H.S., Duhig, T., Nam, M., Palmer, G., et al. (2010). Analysis of a genome-wide set of gene deletions in the fission yeast Schizosaccharomyces pombe. Nat. Biotechnol. *28*, 617–623.

Kim, L., Hoe, K.L., Yu, Y.M., Yeon, J.H., and Maeng, P.J. (2012). The fission yeast GATA factor, Gaf1, modulates sexual development via direct down-regulation of *ste11+* expression in response to nitrogen starvation. PLoS ONE *7*, e42409.

Kuroda, K., Hammer, S.K., Watanabe, Y., Montaño López, J., Fink, G.R., Stephanopoulos, G., Ueda, M., and Avalos, J.L. (2019). Critical roles of the pentose phosphate pathway and GLN3 in isobutanol-specific tolerance in yeast. Cell Syst. *9*, 534–547.e5.

Langmead, B., and Salzberg, S.L. (2012). Fast gapped-read alignment with Bowtie 2. Nat. Methods *9*, 357–359.

Laor, D., Cohen, A., Kupiec, M., and Weisman, R. (2015). TORC1 regulates developmental responses to nitrogen stress via regulation of the GATA transcription factor Gaf1. MBio *6*, e00959.

Leontieva, O.V., and Blagosklonny, M.V. (2016). Gerosuppression by pan-mTOR inhibitors. Aging (Albany N.Y.) *8*, 3535–3551.

Li, H., Handsaker, B., Wysoker, A., Fennell, T., Ruan, J., Homer, N., Marth, G., Abecasis, G., and Durbin, R.; 1000 Genome Project Data Processing Subgroup (2009). The Sequence Alignment/Map format and SAMtools. Bioinformatics *25*, 2078–2079.

Lie, S., Banks, P., Lawless, C., Lydall, D., and Petersen, J. (2018). The contribution of non-essential *Schizosaccharomyces pombe* genes to fitness in response to altered nutrient supply and target of rapamycin activity. Open Biol. *8*, 180015.

Liu, X.M., Sun, L.L., Hu, W., Ding, Y.H., Dong, M.Q., and Du, L.L. (2015). ESCRTs cooperate with a selective autophagy receptor to mediate vacuolar targeting of soluble cargos. Mol. Cell *59*, 1035–1042.

Ma, X.M., and Blenis, J. (2009). Molecular mechanisms of mTOR-mediated translational control. Nat. Rev. Mol. Cell Biol. *10*, 307–318.

Ma, Y., Ma, N., Liu, Q., Qi, Y., Manabe, R., and Furuyashiki, T. (2015). Tor signaling regulates transcription of amino acid permeases through a GATA transcription factor Gaf1 in fission yeast. PLoS ONE *10*, e0144677.

Marguerat, S., Jensen, T.S., de Lichtenberg, U., Wilhelm, B.T., Jensen, L.J., and Bähler, J. (2006). The more the merrier: comparative analysis of microarray studies on cell cycle-regulated genes in fission yeast. Yeast *23*, 261–277.

Marguerat, S., Schmidt, A., Codlin, S., Chen, W., Aebersold, R., and Bähler, J. (2012). Quantitative analysis of fission yeast transcriptomes and proteomes in proliferating and quiescent cells. Cell *151*, 671–683.

Mason, J.S., Wileman, T., and Chapman, T. (2018). Lifespan extension without fertility reduction following dietary addition of the autophagy activator Torin1 in *Drosophila melanogaster*. PLoS ONE *13*, e0190105.

Mata, J., Lyne, R., Burns, G., and Bähler, J. (2002). The transcriptional program of meiosis and sporulation in fission yeast. Nat. Genet. *32*, 143–147.

Matsuo, T., Otsubo, Y., Urano, J., Tamanoi, F., and Yamamoto, M. (2007). Loss of the TOR kinase Tor2 mimics nitrogen starvation and activates the sexual development pathway in fission yeast. Mol. Cell. Biol. *27*, 3154–3164.

Mayer, C., Zhao, J., Yuan, X., and Grummt, I. (2004). mTOR-dependent activation of the transcription factor TIF-IA links rRNA synthesis to nutrient availability. Genes Dev. *18*, 423–434.

McCormick, M.A., Delaney, J.R., Tsuchiya, M., Tsuchiyama, S., Shemorry, A., Sim, S., Chou, A.C.Z., Ahmed, U., Carr, D., Murakami, C.J., et al. (2015). A comprehensive analysis of replicative lifespan in 4,698 single-gene deletion strains uncovers conserved mechanisms of aging. Cell Metab. *22*, 895–906.

Mercier, A., Pelletier, B., and Labbé, S. (2006). A transcription factor cascade involving Fep1 and the CCAAT-binding factor Php4 regulates gene expression in response to iron deficiency in the fission yeast *Schizosaccharomyces pombe*. Eukaryot. Cell *5*, 1866–1881.

Michels, A.A., Robitaille, A.M., Buczynski-Ruchonnet, D., Hodroj, W., Reina, J.H., Hall, M.N., and Hernandez, N. (2010). mTORC1 directly phosphorylates and regulates human MAF1. Mol. Cell. Biol. *30*, 3749–3757.

Moreno, S., Klar, A., and Nurse, P. (1991). Molecular genetic analysis of fission yeast *Schizosaccharomyces pombe*. Methods Enzymol. *194*, 795–823.

Mulleder, M., Calvani, E., Alam, M.T., Wang, R.K., Eckerstorfer, F., Zelezniak, A., and Ralser, M. (2016). Functional Metabolomics Describes the Yeast Biosynthetic Regulome. Cell *167*, 553–565.

Otsubo, Y., Matsuo, T., Nishimura, A., Yamamoto, M., and Yamashita, A. (2018). tRNA production links nutrient conditions to the onset of sexual differentiation through the TORC1 pathway. EMBO Rep. *19*, e44867.

Poüs, C., and Codogno, P. (2011). Lysosome positioning coordinates mTORC1 activity and autophagy. Nat. Cell Biol. *13*, 342–344.

Puria, R., Zurita-Martinez, S.A., and Cardenas, M.E. (2008). Nuclear translocation of Gln3 in response to nutrient signals requires Golgi-to-endosome trafficking in *Saccharomyces cerevisiae*. Proc. Natl. Acad. Sci. USA *105*, 7194–7199.

Rallis, C., and Bähler, J. (2013). Inhibition of TORC1 signaling and increased lifespan: gained in translation? Aging (Albany N.Y.) *5*, 335–336.

Rallis, C., Codlin, S., and Bähler, J. (2013). TORC1 signaling inhibition by rapamycin and caffeine affect lifespan, global gene expression, and cell proliferation of fission yeast. Aging Cell *12*, 563–573.

Rallis, C., López-Maury, L., Georgescu, T., Pancaldi, V., and Bähler, J. (2014). Systematic screen for mutants resistant to TORC1 inhibition in fission yeast reveals genes involved in cellular ageing and growth. Biol. Open *3*, 161–171.

Ramírez, F., Ryan, D.P., Grüning, B., Bhardwaj, V., Kilpert, F., Richter, A.S., Heyne, S., Dündar, F., and Manke, T. (2016). deepTools2: a next generation web server for deep-sequencing data analysis. Nucleic Acids Res. *44* (*W1*), W160–W165.

Raudvere, U., Kolberg, L., Kuzmin, I., Arak, T., Adler, P., Peterson, H., and Vilo, J. (2019). g:Profiler: a web server for functional enrichment analysis and conversions of gene lists (2019 update). Nucleic Acids Res. *47* (*W1*), W191–W198.

Roux, A.E., Quissac, A., Chartrand, P., Ferbeyre, G., and Rokeach, L.A. (2006). Regulation of chronological aging in *Schizosaccharomyces pombe* by the protein kinases Pka1 and Sck2. Aging Cell *5*, 345–357.

Rustici, G., Mata, J., Kivinen, K., Lió, P., Penkett, C.J., Burns, G., Hayles, J., Brazma, A., Nurse, P., and Bähler, J. (2004). Periodic gene expression program of the fission yeast cell cycle. Nat. Genet. *36*, 809–817.

Saxton, R.A., and Sabatini, D.M. (2017). mTOR signaling in growth, metabolism, and disease. Cell *169*, 361–371.

Scherens, B., Feller, A., Vierendeels, F., Messenguy, F., and Dubois, E. (2006). Identification of direct and indirect targets of the Gln3 and Gat1 activators by transcriptional profiling in response to nitrogen availability in the short and long term. FEMS Yeast Res. *6*, 777–791.

Schindelin, J., Arganda-Carreras, I., Frise, E., Kaynig, V., Longair, M., Pietzsch, T., Preibisch, S., Rueden, C., Saalfeld, S., Schmid, B., et al. (2012). Fiji: an open-source platform for biological-image analysis. Nat. Methods *9*, 676–682.

Schneider, C.A., Rasband, W.S., and Eliceiri, K.W. (2012). NIH Image to ImageJ: 25 years of image analysis. Nat. Methods *9*, 671–675.

Selman, C., Tullet, J.M.A., Wieser, D., Irvine, E., Lingard, S.J., Choudhury, A.I., Claret, M., Al-Qassab, H., Carmignac, D., Ramadani, F., et al. (2009). Ribosomal protein S6 kinase 1 signaling regulates mammalian life span. Science *326*, 140–144.

Shimanuki, M., Uehara, L., Pluskal, T., Yoshida, T., Kokubu, A., Kawasaki, Y., and Yanagida, M. (2013). Klf1, a C2H2 zinc finger-transcription factor, is required for cell wall maintenance during long-term quiescence in differentiated G0 phase. PLoS ONE *8*, e78545.

Shor, B., Wu, J., Shakey, Q., Toral-Barza, L., Shi, C., Follettie, M., and Yu, K. (2010). Requirement of the mTOR kinase for the regulation of Maf1 phosphorylation and control of RNA polymerase III-dependent transcription in cancer cells. J. Biol. Chem. *285*, 15380–15392.

Thoreen, C.C., Kang, S.A., Chang, J.W., Liu, Q., Zhang, J., Gao, Y., Reichling, L.J., Sim, T., Sabatini, D.M., and Gray, N.S. (2009). An ATP-competitive mammalian target of rapamycin inhibitor reveals rapamycin-resistant functions of mTORC1. J. Biol. Chem. *284*, 8023–8032.

Valbuena, N., Guan, K.L., and Moreno, S. (2012). The Vam6 and Gtr1-Gtr2 pathway activates TORC1 in response to amino acids in fission yeast. J. Cell Sci. *125*, 1920–1928.

Valvezan, A.J., and Manning, B.D. (2019). Molecular logic of mTORC1 signalling as a metabolic rheostat. Nat. Metabolism *1*, 321–333.

Viger, R.S., Guittot, S.M., Anttonen, M., Wilson, D.B., and Heikinheimo, M. (2008). Role of the GATA family of transcription factors in endocrine development, function, and disease. Mol. Endocrinol. *22*, 781–798.

Wamaitha, S.E., del Valle, I., Cho, L.T.Y., Wei, Y., Fogarty, N.M.E., Blakeley, P., Sherwood, R.I., Ji, H., and Niakan, K.K. (2015). Gata6 potently initiates reprograming of pluripotent and differentiated cells to extraembryonic endoderm stem cells. Genes Dev. *29*, 1239–1255.

Wei, Y., Tsang, C.K., and Zheng, X.F.S. (2009). Mechanisms of regulation of RNA polymerase III-dependent transcription by TORC1. EMBO J. *28*, 2220–2230.

Wei, Y., Zhang, Y.J., and Cai, Y. (2013). Growth or longevity: the TOR's decision on lifespan regulation. Biogerontology *14*, 353–363.

Wei, Y., and Zheng, X.S. (2010). Maf1 regulation: a model of signal transduction inside the nucleus. Nucleus *1*, 162–165.

Weisman, R., and Choder, M. (2001). The fission yeast TOR homolog, *tor1*$^{+}$, is required for the response to starvation and other stresses via a conserved serine. J. Biol. Chem. *276*, 7027–7032.

Wilkinson, M.G., Samuels, M., Takeda, T., Toone, W.M., Shieh, J.C., Toda, T., Millar, J.B., and Jones, N. (1996). The Atf1 transcription factor is a target for the Sty1 stress-activated MAP kinase pathway in fission yeast. Genes Dev. *10*, 2289–2301.

Xie, Y., Jin, Y., Merenick, B.L., Ding, M., Fetalvero, K.M., Wagner, R.J., Mai, A., Gleim, S., Tucker, D.F., Birnbaum, M.J., et al. (2015). Phosphorylation of GATA-6 is required for vascular smooth muscle cell differentiation after mTORC1 inhibition. Sci. Signal. *8*, ra44.

Zhong, Y., Wang, Z., Fu, B., Pan, F., Yachida, S., Dhara, M., Albesiano, E., Li, L., Naito, Y., Vilardell, F., et al. (2011). GATA6 activates Wnt signaling in pancreatic cancer by negatively regulating the Wnt antagonist Dickkopf-1. PLoS ONE *6*, e22129.

Zhou, D., Zhong, S., Ye, J.J., Quach, K.M., Johnson, D.L., and Chen, S. (2007). PNRC is a unique nuclear receptor coactivator that stimulates RNA polymerase III-dependent transcription. J. Mol. Signal. *2*, 5.

Zhu, L.J., Gazin, C., Lawson, N.D., Pagès, H., Lin, S.M., Lapointe, D.S., and Green, M.R. (2010). ChIPpeakAnno: a Bioconductor package to annotate ChIP-seq and ChIP-chip data. BMC Bioinformatics *11*, 237.

Zilio, N., Codlin, S., Vashisht, A.A., Bitton, D.A., Head, S.R., Wohlschlegel, J.A., Bähler, J., and Boddy, M.N. (2014). A novel histone deacetylase complex in the control of transcription and genome stability. Mol. Cell. Biol. *34*, 3500–3514.

STAR★METHODS

KEY RESOURCES TABLE

REAGENT or RESOURCE	SOURCE	IDENTIFIER
Antibodies		
Phospho-(Ser/Thr) Akt Substrate (PAS)	Cell Signaling	Cat#9611; RRID:AB_330302
Anti-rps6	Abcam	Cat#ab40820; RRID:AB_945319
anti-rabbit HRP	Abcam	Cat#ab6721; RRID:AB_955447
Anti-GFP	Abcam	Cat#ab290; RRID:AB_303395
Anti-HA	Abcam	Cat#ab9110; RRID:AB_307019
Dynabeads M-280 sheep anti Rabbit IgG	Thermo Fisher	Cat# 11203D; RRID:AB_2783009
Chemicals, Peptides, and Recombinant Proteins		
Rapamycin	LC Laboratories	# R-5000
Caffeine	Sigma	# 27602-250G
Torin1	TOCRIS	#4247
FM4-64	ThermoFisher	#T13320
Calcuofluor	Sigma	#18909
Doxycycline hyclate	Sigma	# D9891
CdSO4	Sigma	# 202924
Cycloheximide	Sigma	# C7698
PMSF	Sigma	#10837091001
Phosphatase Inhibitor Cocktail 1	Sigma	#P2850
Phosphatase Inhibitor Cocktail 2	Sigma	#P5726
Complete, EDTA free protease Inhibitor Cocktail	Merck	#11873580001
Critical Commercial Assays		
Microarrays	Agilent	Custom design 8x15K
NEBNext® ultra DNA Library Prep kit	New England Biolabs	E7370L
ECL Western Blotting Detection System	GE Healthcare	GERPN2134
DIG Oligonucleotide 3′ End Labeling Kit, 2nd generation	Merk	#03353575910
DIG Luminescent Detection Kit	Merk	# 11363514910
DIG Wash and Block Buffer Set	Merk	# 11585762001
BrightStar-Plus Positively Charged Nylon Membrane	Thermo Fisher	# AM10100
Mini-PROTEAN® TBE-Urea Precast Gels	Bio Rad	# 4566036
MiSeq Reagent Kit v3 (150-cycle)	Illumina	MS-102-3001
Deposited Data		
ChIP-seq data	ENA	Accession numbers: PRJEB32910 and ERP115647
Microarray data	ArrayExpress	Accession number: E-MTAB-8569
Experimental Models: Organisms/Strains		
Fission yeast 972^{-}	Bahler lab strainlist	JB903
Fission yeast Gaf1-GFP	Bahler lab strainlist	JB1744
Fission yeast Bioneer strains	Bioneer	N/A
Oligonucleotides		
Cdc-SRT	cdc2-SRT GGGCAGGGTCATAAACAAGC	Clément-Ziza et al., 2014
tRNA-leu-CAA-intron-5	GACTATCGTCCAAGTATTACTTGAGTGCTGCG	Otsubo et al., 2018

(Continued on next page)

Continued

REAGENT or RESOURCE	SOURCE	IDENTIFIER
tRNA-asn01-5leader	TATGCTACCCGACCTATAATGCTCCTGGTGAG	Otsubo et al., 2018
Software and Algorithms		
ImageJ	Schneider et al., 2012	https://imagej.nih.gov/ij/
Volocity acquisition program	PerkinElmer	https://www.perkinelmer.com/
Volocity quantitation package	PerkinElmer	https://www.perkinelmer.com/
GEM	Guo et al., 2012	http://groups.csail.mit.edu/cgs/gem/
Bowtie2	Langmead and Salzberg, 2012	http://bowtie-bio.sourceforge.net/bowtie2/index.shtml
ChIPpeakAnno	Zhu et al., 2010	https://www.bioconductor.org/packages/release/bioc/html/ChIPpeakAnno.html
Deeptools	Ramírez et al., 2016	https://deeptools.readthedocs.io/en/develop/
Samtools	Li et al., 2009	http://samtools.sourceforge.net/

LEAD CONTACT AND MATERIALS AVAILABILITY

Further information and requests for resources and reagents should be directed to and will be fulfilled by the Lead Contact, Jürg Bähler (j.bahler@ucl.ac.uk). This study generated new *S. pombe* strains that are available from the Lead Contact without restriction upon request.

EXPERIMENTAL MODEL AND SUBJECT DETAILS

This study has been conducted using *S. pombe* as experimental model. For wild-type control strains, we used *972 h⁻* or the parental strains for the deletion library, ED666 (*h+ ade6-M210 ura4-D18 leu1–32*) and ED668 (*h+ ade6- M216 ura4-D18 leu1–32*). The Bioneer haploid deletion library strains used for further studies were PCR-validated and backcrossed with *972 h⁻*. The *gaf1-GFP* strain was generated as described (Bahler et al., 1998). Cell cultures were grown in yeast extract plus supplements (YES) as default or in Edinburgh minimal medium (EMM2) if indicated (Moreno et al., 1991). Liquid cultures were grown at 32°C with shaking at 130 rotations per minute.

METHOD DETAILS

Drug sensitivity assays

Cells were grown in liquid YES to an OD_{600} of 0.5. Ten-fold serial dilutions of cells were spotted onto YES agar plates, using replica platers for 48-well or 96-well plates (Sigma), with or without drugs as indicated in figure legends.

Measurement of cell size and fluorescence microscopy

To determine cell size, control and drug-treated cells were fixed in 4% formaldehyde for 10 min at room temperature, washed with 50 mM sodium citrate, 100 mM sodium phosphate, and stained with Calcofluor (50 mg/ml). Microscopy was performed using a DAPI filter for Calcofluor detection and a Hamamatsu ORCA-ER C4742-95 digital camera fitted to a Zeiss Axioskop microscope with EC plan-NEOFLUAR 63x 1.25 NA oil objective. Images were recorded using the Volocity acquisition program (PerkinElmer). At least 100 septated cells were counted and analyzed for each condition using the Volocity quantitation package (PerkinElmer). Results were analyzed in R. For fluorescence microscopy of Gaf1-GFP cells, we used a spinning disk confocal microscope (Yokogawa CSU-X1 head mounted on Olympus body; CoolSnap HQ2 camera [Roper Scientific], Plan Apochromat 100X, 1.4 NA objective [Olympus]). The images correspond to maximum intensity projections of 15 image stacks with a Z-step of 0.3 microns. Cells were immobilized with soybean lectin (Sigma L1395) in two different compartments of a glass-bottom 15 μ-Slide 8 well (Ibidi 80821) to add either DMSO as a solvent control or Torin1 (to a final concentration of 20 μM, dissolved in DMSO). *In vivo* chromatin staining was done with Hoechst 33342 (1 μg/ml). As this dye performs poorly in YES, cells were immobilized onto glass bottom wells and washed three times with liquid EMM2 containing Hoechst 33342 (Sigma-Aldrich B2261) at 1 μg/ml plus Torin1 (20 μM). Cells were covered with this media and imaged 10 min later. Image analysis and editing was performed using Fiji (ImageJ) open software (Schindelin et al., 2012).

Measurement of vacuolar size

Vacuolar labeling was performed as described (Codlin and Mole, 2009). Briefly, FM4-64 dye (Molecular Probes) was dissolved in DMSO at a concentration of 0.82 mM, and 2 μL FM4-64 stock was added to 1 mL log-phase cells with or without drugs. Following

30 min exposure to FM4-64, cells were washed and chased for 40 min in fresh media to allow all dye to reach the vacuole. Fluorescence microscopy was performed using a Rhodamine filter for detection of FM4-64 and a Hamamatsu ORCA-ER C4742-95 digital camera fitted to a Zeiss Axioskop microscope with EC plan-NEOFLUAR × 63 1.25 NA oil objective. Images were recorded using the Volocity acquisition program (PerkinElmer). At least 500 vacuoles were measured using the Volocity quantitation package (PerkinElmer). Results were analyzed in R.

Chronological lifespan assay

Cells were grown in EMM2 media as described (Rallis et al., 2013). When cultures reached a stable maximal density, cells were left an additional 24 hr and then harvested, serially diluted, and incubated on YES plates. The measurement of colony-forming units (CFUs) was taken at the beginning of the lifespan curve (time point 0: 100% cell survival). CFU measurements were conducted on successive days until cultures dropped to 0.1% cell survival. Error bars represent standard deviation calculated from three independent cultures, with each culture measured three times at each time point. To determine the chronological lifespan when TOR is inhibited, 8 μM Torin1 was added to rapidly proliferating cell cultures at OD_{600} = 0.5 which were then grown to stationary phase, and lifespan was recorded as described above. AUCs were measured with ImageJ (Schindelin et al., 2012) for all experimental repeats using lifespans curves on the linear scale for % survival.

High-throughput genetic screening

The haploid deletion libraries were plated onto YES plates containing 100 μg/ml G418 using a RoToR HDA robot (Singer). Multiple replicate copies of the library were thus generated. Using the RoToR, the libraries were compacted into nine 384-density plates of plates and then printed onto plates containing 20 μM Torin1. The plates were incubated at 32°C for 2 days and then manually scored for resistant colonies.

Growth assay

Growth in the presence or absence of Torin1 were automatically determined in 48-well flowerplates at 1.5 mL volumes, 1000 rpm and 32°C using the Biolector microfermentation system (m2p-biolabs). Growth dynamics were modeled using the grofit R package (Kahm et al., 2010). In the resulting growth curves, the units of the x axis are time (hr) while the y axis shows biomass (arbitrary units) normalized to biomass at time 0.

Western blotting and antibodies

For protein preparations, cells were diluted in 6 mM Na_2HPO_4, 4mM $NaH_2PO_4.H_2O$, 1% Nonidet P-40, 150 mM NaCl, 2 mM EDTA, 50 mM NaF supplemented with protease (PMSF) and phosphatase inhibitors (Sigma cocktails 1 and 2), together with glass beads. Cells were lysed in a Fastprep-24 machine (MP Biomedicals). Phospho-(Ser/Thr) Akt Substrate (PAS) Antibody (9611, Cell Signaling) for detection of P-S6 (p27) and anti-rps6 (ab40820, Abcam) were used at 1/2000 dilution. For detection, we used the anti-rabbit HRP-conjugated antibody (1/5000 dilutions) with the ECL Western Blotting Detection System (GE Healthcare) according to the manufacturer's protocol.

Microarrays

Microarray analysis was performed as previously described (Rallis et al., 2013). Cells were grown in YES to OD_{600} = 0.5 and harvested. Torin1 treatments were done for 1 hr at a concentration of 20 μM. Caffeine/rapamycin treatments were also performed for 1 hr at concentrations 10mM caffeine and 100ng/ml rapamycin. Two independent biological repeats with a dye swap were performed. For each repeat, a corresponding pool of Torin1 or caffeine/rapamycin treated and untreated wild-type and *gaf1Δ* cells was used as a common reference for microarray hybridization. Agilent 8 × 15K custom-made *S. pombe* expression microarrays were used, with hybridizations and subsequent washes performed according to the manufacturer's protocols. The microarrays were scanned and extracted using GenePix (Molecular Devices), processed using R scripts for quality control and normalization, and analyzed using GeneSpring GX3 (Agilent). We determined genes that were 1.5-fold upregulated or downregulated in both repeats of Torin1-treated and caffeine/rapamycin-treated *gaf1Δ* cells relative to Torin1-treated and caffeine/rapamycin-treated wild-type cells respectively.

ChIP-seq

Cells were grown in YES to an OD_{600} of ~0.4. Untreated and Torin 1-treated (20 μM for 15 min or 1 hr) cells were fixed in 1% formaldehyde for 30 min and then quenched 10 min with 125mM glycine. Pellets were washed with ice-cold PBS, snap frozen in liquid nitrogen and stored at −80°C. Cell pellets were resuspended in lysis buffer (50 mM HEPES pH 7.6, 1mM EDTA pH 8, 150 mM NaCl, 1% Triton X-100, 0.1% sodium doxycolate, 1mM PMSF and protease inhibitors). Chromatin was obtained following cell disruption using a Fastprep-24 (MP Biomedicals) and sheared using a Bioruptor (Diagenode). Dynabeads M-280 sheep anti-rabbit IgG were incubated in lysis buffer and 0.5% BSA for 2 hr with either rabbit anti-GFP (Abcam) for query IPs or 5 μl of rabbit-anti HA (Abcam) for control IPs. Then, 2 mg of Chromatin extract were inmunoprecipitated for 16 hr using the corresponding antibody-incubated Dynabeads. Following the washes, DNA was eluted, treated with RNase and proteinase K, and purified using the QIAGEN PCR MiniElute kit. Sequencing libraries were prepared using the NEBNext® ultra DNA Library Prep kit for Illumina® (E7370L). DNA

was sequenced using Illumina Mi-seq with a V3 kit, sequencing 75 bp on each end. Sequences were aligned to the *S. pombe* genome build ASM294v2 using Bowtie2. Peak calling was done with GEM (Guo et al., 2012) (setting–k_min 4 and–k_max 18), and peak annotation was done with the R package ChIPpeakAnno (Zhu et al., 2010). Peaks were annotated to the closest TSS; for peaks lying within 500 bp of 2 divergently expressed genes, peaks were annotated to both genes. Normalizations for the plots were performed using deeptools (Ramírez et al., 2016) (normalizing to RPGC and using the parameters –centerReads –binsize 10 –smoothLength 2). Further analyses were carried out with R scripts (http://www.r-project.org/). Gene enrichment analysis was performed using AnGeLi (Bitton et al., 2015) and g:profiler (Raudvere et al., 2019).

Northern analyses

Detection of tRNA precursors was performed as described (Otsubo et al., 2018) using Digoxigenin labeled probes (Roche), following the manufacturer's instructions. As a loading control, northerns were stripped by incubating for 60 min at 60°C with 0.1% SDS, changing the solution every 10 min, followed by re-hybridizing with a Digoxigenin labeled probe specific for *cdc2* (cdc2-SRT GGGCAGGGTCATAAACAAGC) as described (Clément-Ziza et al., 2014). Quantification of Northern blots has been performed by ImageJ (Schindelin et al., 2012) as previously described (Rallis et al., 2014). Ratios of each tRNA band signal with the corresponding *cdc2* loading control have been normalized with the ratio at time point 0 for each tRNA and genotype.

QUANTIFICATION AND STATISTICAL ANALYSIS

Northern blot and lifespan AUC quantification has been conducted using ImageJ using 3 independent biological repeats (n = 3). Significance has been defined using t tests with a p value cutoff of 0.05. Microarray experiments have been conducted in 2 biological repeats with a dye swap. ChIP-seq experiments have been conducted in 2 biological repeats. Quantifications and statistical analysis are also described in corresponding STAR Methods sections. Lifespan assays have been performed in three biological repeats with each time point measured in three technical repeats for each biological replicate. t tests were used for AUC statistics. For Figures S3 and S4, p values refer to adjusted p values using the g:SCS algorithm described in the g:profiler software (Raudvere et al., 2019). For Figure 1B, each boxplot represents at least 500 measured vacuoles. In Figure S1, each boxplot represents at least 100 measured cells.

DATA AND CODE AVAILABILITY

Scripts for Analysis

Scripts, packages and programs used for analyses are mentioned in the main text and listed within the Key Resources Table.

Dataset Hosting

The accession numbers for the ChIP-seq data reported in this paper are ENA: PRJEB32910 and ERP115647. The accession number for the microarray data reported in this paper is ArrayExpress: E-MTAB-8569.

Cell Reports, Volume *30*

Supplemental Information

The GATA Transcription Factor Gaf1 Represses tRNAs, Inhibits Growth, and Extends Chronological Lifespan Downstream of Fission Yeast TORC1

María Rodríguez-López, Suam Gonzalez, Olivia Hillson, Edward Tunnacliffe, Sandra Codlin, Victor A. Tallada, Jürg Bähler, and Charalampos Rallis

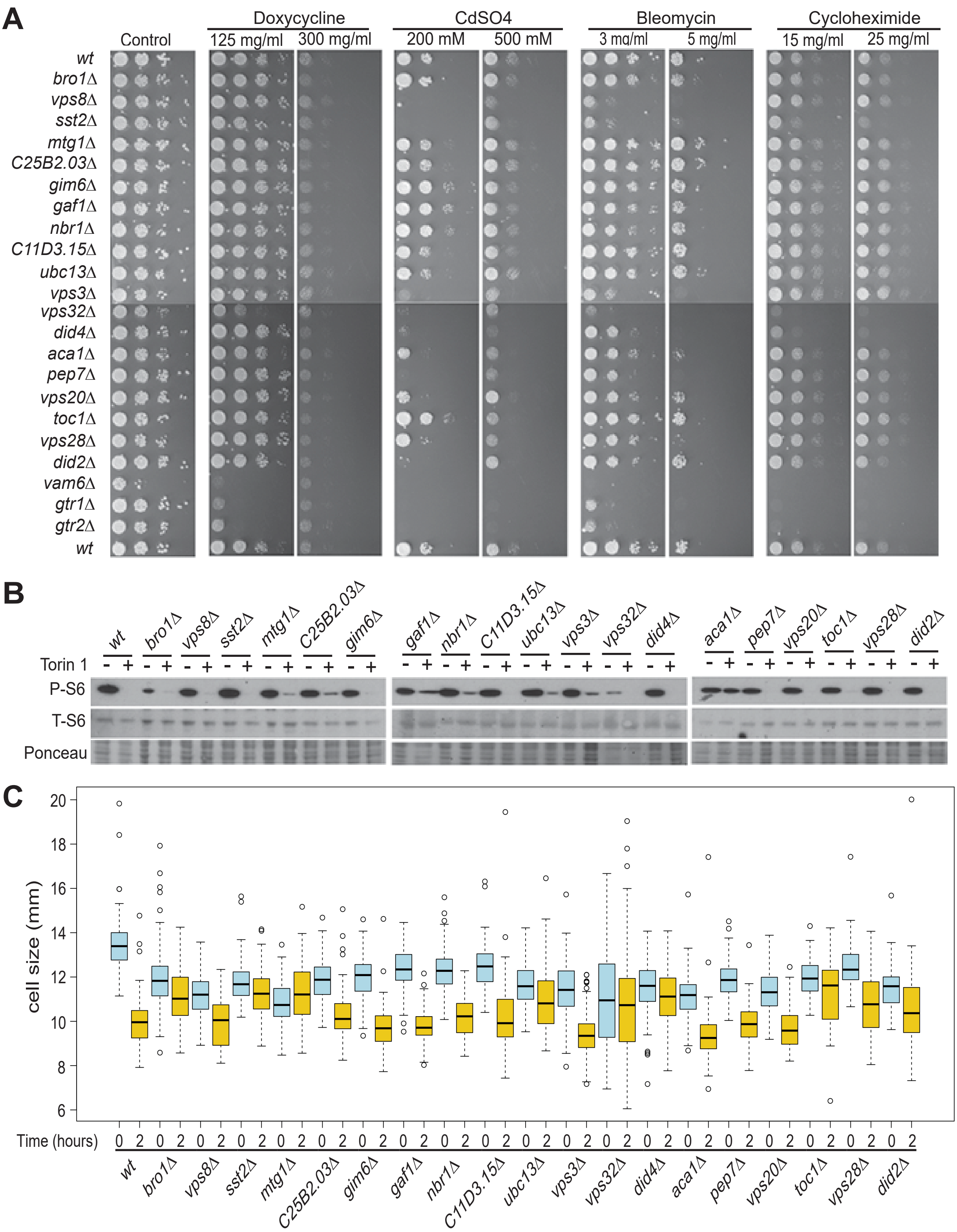

Figure S1. Characterization of Torin1-resistant strains, Related to Figure 1.
A. Resistance to Torin1 is not a result of multi-drug resistance. Spot assays of wild-type and the 19 resistant mutants in presence of different drugs as indicated. Control mutants that show multi-drug sensitivity are also spotted (*vam6Δ*, *gtr1Δ*, *gtr2Δ*) to show that the drugs are functional.
B. Phosphorylation status of ribosomal S6 protein in verified Torin1-resistant mutants in the presence or absence of Torin1 as indicated. **C.** Cell size upon division of wild-type and resistant mutant cells, before (blue) and after (yellow) Torin1 treatment.

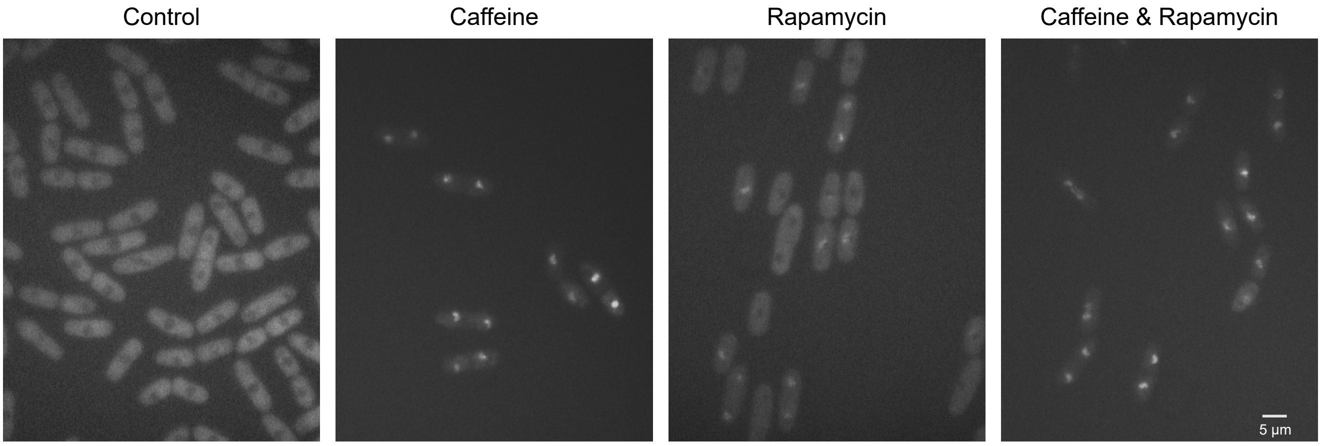

Figure S2. Fluorescence microscopy of cells expressing GFP-tagged Gaf1, Related to Figure 3.
Cells were grown in YES liquid medium to mid-exponential phase (OD600 =0.5), when caffeine (10 mM), rapamycin (100 ng/ml), or both drugs combined at same doses were added, and cells were incubated with shaking at 32°C for 30 minutes. Control cells were not exposed to the drugs but otherwise treated the same. Cells were photographed in a Zeiss Axioskop microscope using the Volocity software. All cells showed nuclear localization of Gaf1 after treatment with caffeine or with both drugs; the nuclear translocation of Gaf1 was less efficient in cells treated with rapamycin only, reflecting that rapamycin alone only partially inhibits TORC1 in *S. pombe* (Rallis et al., 2013).

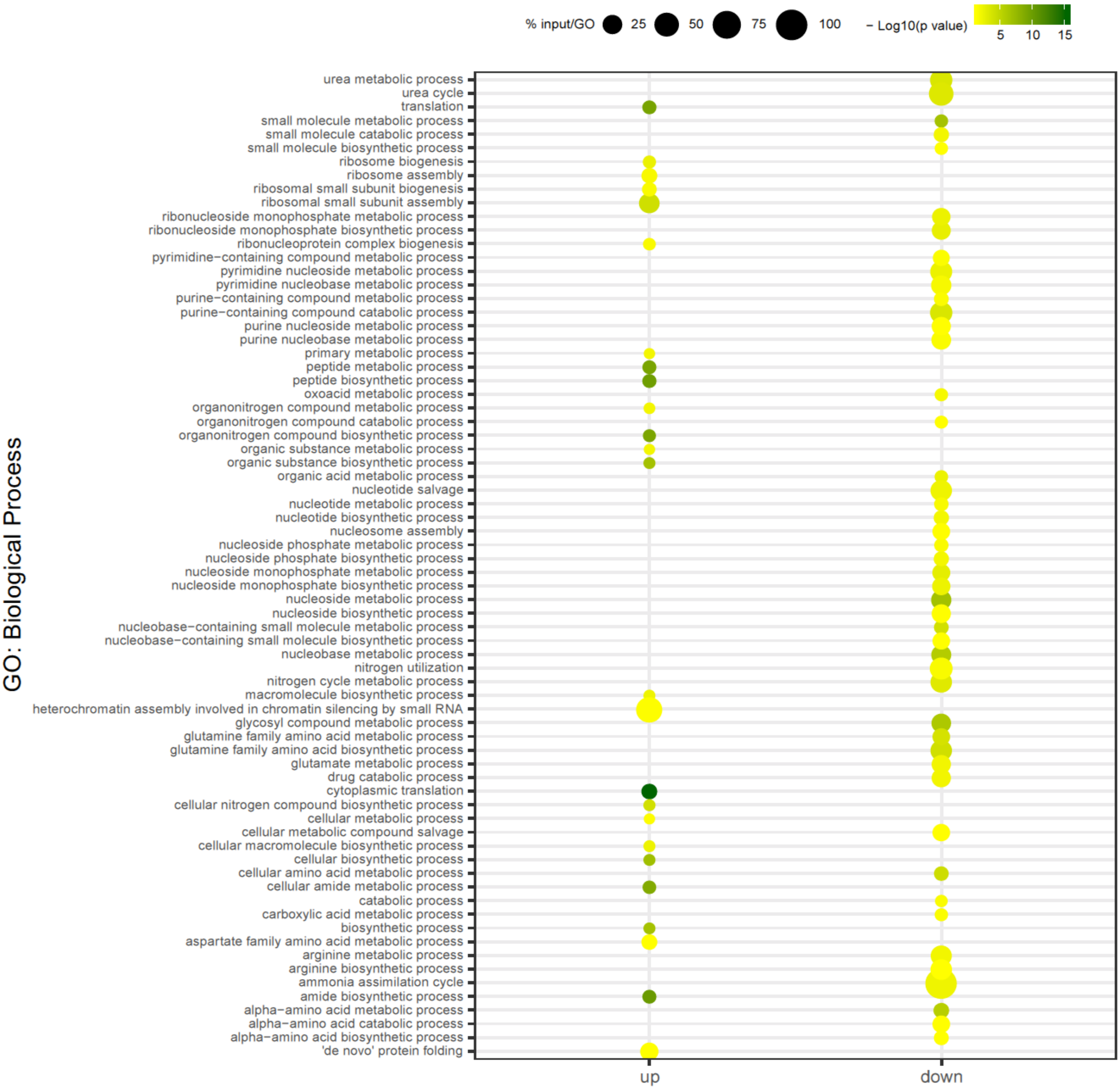

Figure S3. Functional enrichments among differentially expressed genes, Related to Figure 3. Visualization of all GO Biological Process categories enriched among the 108 genes that are repressed (down) and the 90 genes that are induced (up). Gene enrichment analysis was done using g:profiler (https://academic.oup.com/nar/advance-article/doi/10.1093/nar/gkz369/5486750). Results were plotted using a custom R script. Colors represent -log10 p values for the GO term enrichments. The size of the bubbles represents the ratio between the number of genes in the gene list and the total number of genes in the GO category (in percentage); only terms with p values <0.05 are represented.

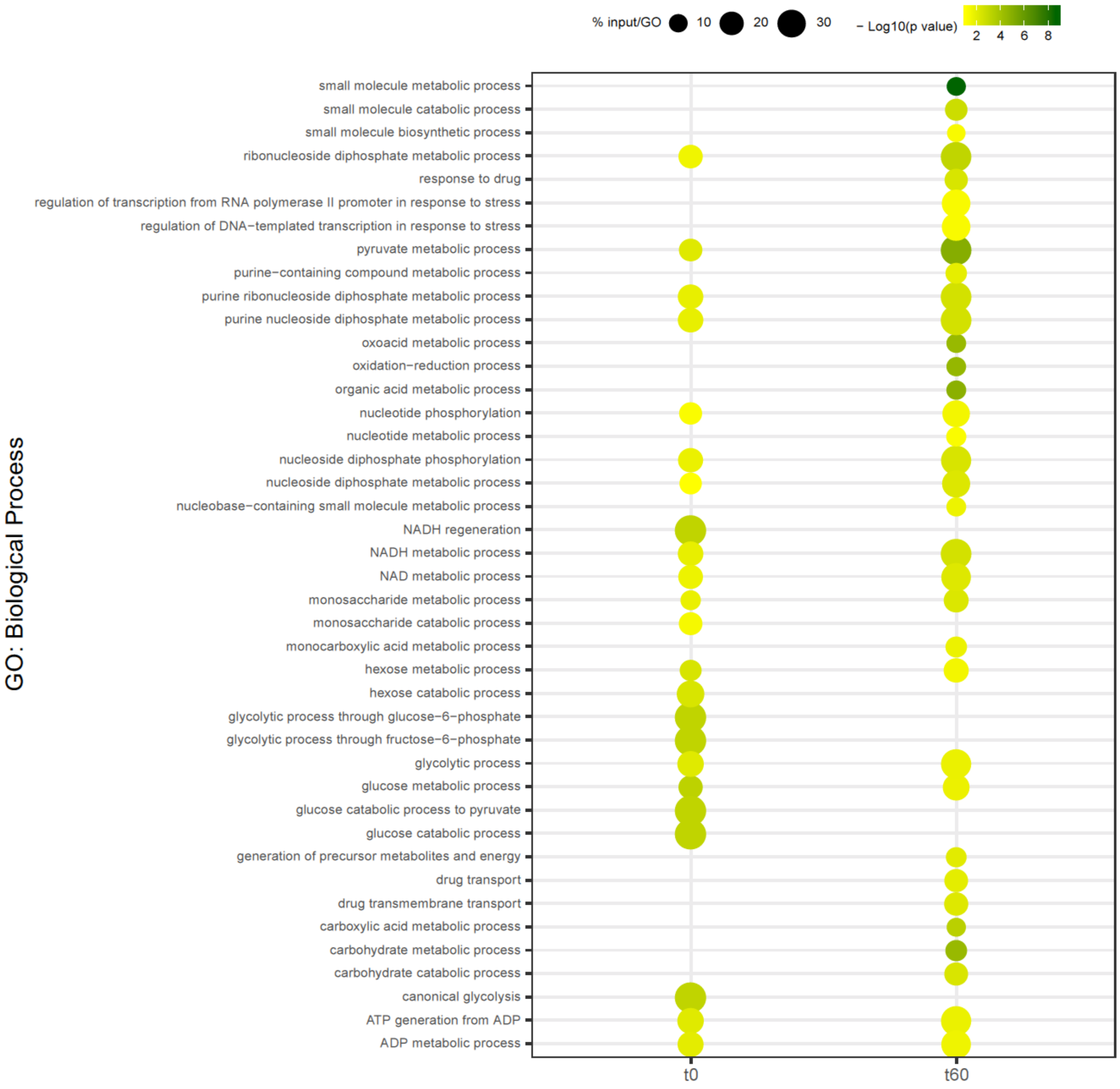

Figure S4. Functional enrichments among Gaf1-target genes, Related to Figure 4.
Visualization of all GO Biological Process categories enriched among the 245 protein-coding genes whose promoters are bound by Gaf1 after 60 min of Torin1 treatment. See Figure S3 for details.

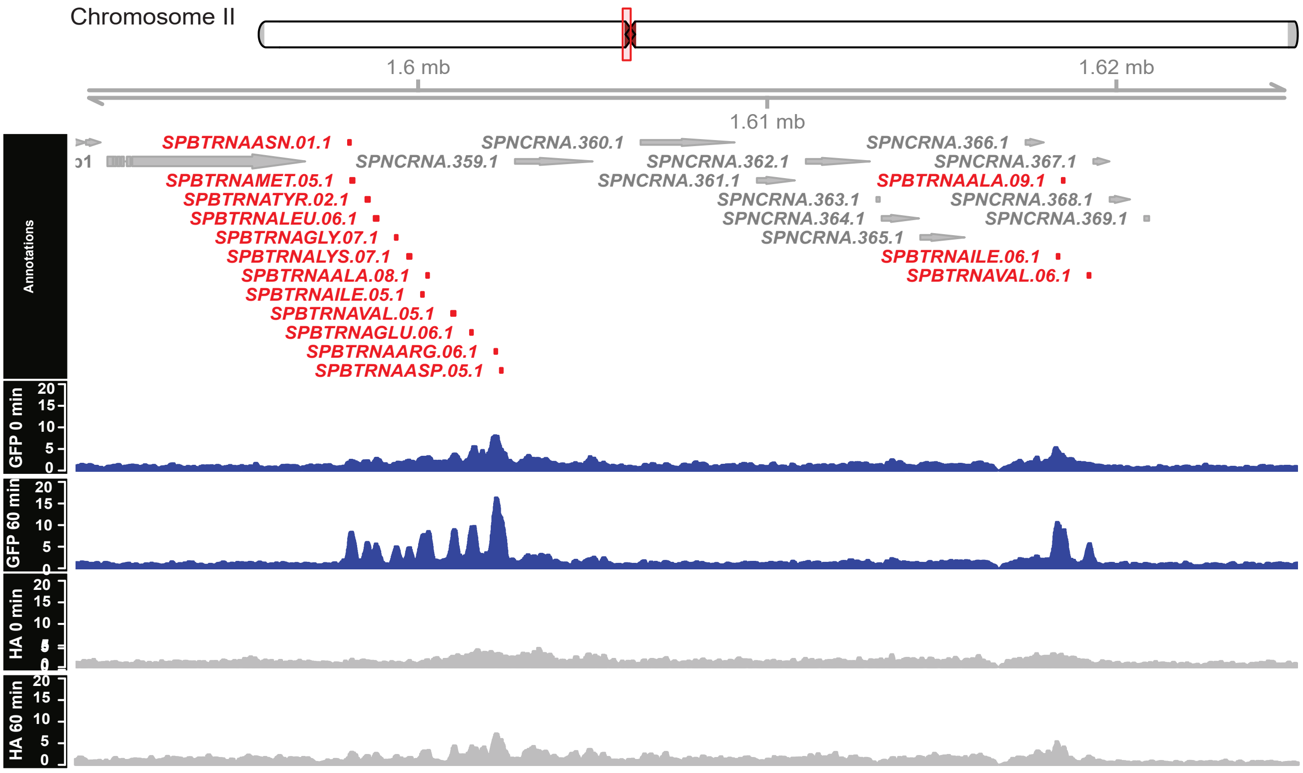

Figure S5. Gaf1 binding at tRNA genes, Related to Figure 4C.
Gaf1 binding peaks within Chromosome II region containing clustered tRNA genes (marked in red). Binding profiles are shown for cells before (0 min) and 60 min after treatment with Torin 1 as indicated. Signals from experimental IPs (GFP) are shown in blue, while control IPs (HA) are shown in grey.

Table S1. Torin1-resistant mutants, Related to Figure 1.
List of the 19 mutants that show resistance to Torin1. Mutants with black fonts have previously been shown to be resistant to Torin1. Mutants with red fonts are first reported as Torin1-resistant in this study.

SYSTEMATIC ID	GENE NAME	PRODUCT DESCRIPTION
SPAC21E11.04	*aca1*	L-azetidine-2-carboxylic acid acetyltransferase Aca1
SPAC17G6.05c	*bro1*	BRO1 domain protein Bro1 (predicted)
SPBC13G1.12	*did2*	ESCRT III complex subunit Did2 (predicted)
SPAC4F8.01	*did4*	ESCRT III complex subunit Did4
SPCC1902.01	*gaf1*	transcription factor Gaf1
SPBC1D7.01	*gim6*	prefoldin subunit 1 (predicted)
SPBC25B2.04c	*mtg1*	mitochondrial translation factor (GTPase) Mtg1 (predicted)
SPBP35G2.11c	*nbr1*	cargo receptor for selective autophagy pathway
SPAC17G6.08	*pep7*	prevacuole/endosomal FYVE tethering component Pep7 (predicted)
SPAC19B12.10	*sst2*	human AMSH/STAMBP protein homolog, ubiquitin specific-protease
SPBP18G5.03	*toc1*	Tor complex Tor2 interacting protein 1
SPAC11E3.04c	*ubc13*	ubiquitin conjugating enzyme E2 Ubc13
SPBC215.14c	*vps20*	ESCRT III complex subunit Vps20
SPAC1B3.07c	*vps28*	ESCRT I complex subunit Vps28
SPCC364.05	*vps3*	CORVET complex subunit, GTPase regulator Vps3 (predicted)
SPAC1142.07c	*vps32*	ESCRT III complex subunit Vps32
SPAC17A2.06c	*vps8*	CORVET complex WD repeat Vps8 (predicted)
SPAC11D3.15		5-oxoprolinase (ATP-hydrolizing) (predicted)
SPBC25B2.03		zf-C3HC4 type zinc finger
